# Melatonin: Regulation of Biomolecular Condensates in Neurodegenerative Disorders

**DOI:** 10.3390/antiox10091483

**Published:** 2021-09-17

**Authors:** Doris Loh, Russel J. Reiter

**Affiliations:** 1 Independent Researcher, Marble Falls, TX 78654, USA; 2 Department of Cellular and Structural Biology, UT Health Science Center, San Antonio, TX 78229, USA

**Keywords:** melatonin, biomolecular condensate, neurodegenerative disorder, liquid–liquid phase separation, ATP, lipid raft, post-translational modification, m^6^A, RNA

## Abstract

Biomolecular condensates are membraneless organelles (MLOs) that form dynamic, chemically distinct subcellular compartments organizing macromolecules such as proteins, RNA, and DNA in unicellular prokaryotic bacteria and complex eukaryotic cells. Separated from surrounding environments, MLOs in the nucleoplasm, cytoplasm, and mitochondria assemble by liquid–liquid phase separation (LLPS) into transient, non-static, liquid-like droplets that regulate essential molecular functions. LLPS is primarily controlled by post-translational modifications (PTMs) that fine-tune the balance between attractive and repulsive charge states and/or binding motifs of proteins. Aberrant phase separation due to dysregulated membrane lipid rafts and/or PTMs, as well as the absence of adequate hydrotropic small molecules such as ATP, or the presence of specific RNA proteins can cause pathological protein aggregation in neurodegenerative disorders. Melatonin may exert a dominant influence over phase separation in biomolecular condensates by optimizing membrane and MLO interdependent reactions through stabilizing lipid raft domains, reducing line tension, and maintaining negative membrane curvature and fluidity. As a potent antioxidant, melatonin protects cardiolipin and other membrane lipids from peroxidation cascades, supporting protein trafficking, signaling, ion channel activities, and ATPase functionality during condensate coacervation or dissolution. Melatonin may even control condensate LLPS through PTM and balance mRNA- and RNA-binding protein composition by regulating N^6^-methyladenosine (m^6^A) modifications. There is currently a lack of pharmaceuticals targeting neurodegenerative disorders via the regulation of phase separation. The potential of melatonin in the modulation of biomolecular condensate in the attenuation of aberrant condensate aggregation in neurodegenerative disorders is discussed in this review.

## 1. Introduction

Present in all cells, biomolecular condensates are membraneless organelles (MLOs) containing proteins, ribonucleic acids (RNAs), and other nucleic acids [1]. These micron-scale macromolecules that can assemble into liquid-like droplets have been proposed to be the origin of life [2]. Current cell and molecular biology reveal that liquid–liquid phase separation (LLPS) is the driving force behind the assembly or dissolution of biomolecules in energy-efficient, rapid, essential reactions to changing endogenous or exogenous conditions including stress response [3] and signal transduction [4,5], as well as genome expression, organization, and repair [6]. LLPS creates distinct compartments that enhance or restrict biochemical reactions by enriching or excluding biomolecules from their environment [7]. Increasing evidence associates diseases such as neurodegeneration and cancer with the formation of protein aggregates from dysregulated, aberrant transitions in phase separation [8,9,10,11,12].

Phase separation at its core is a thermodynamic process driven by the reduction or a negative change in global free energy [1,13]. LLPS is entropically unfavorable; therefore, multivalent protein–protein interactions that are energetically favorable may be necessary to offset energetic costs [14]. Adenosine triphosphate (ATP) is the molecule favored by most organisms for capturing and transferring free energy. During hydrolysis, ATP is transformed into adenosine diphosphate (ADP) and inorganic phosphate (Pi). The change in free energy of −7.3 kcal/mol associated with this chemical reaction is used by cells to perform energetically favorable reactions [15], including relevant post-translational modification (PTM) such as phosphorylation [16], ubiquitination [17,18], and SUMOylation that may regulate condensate nucleation, composition, and growth [19,20]. It is understood that most proteins in the human proteome can undergo LLPS, assembling into dense liquid-like, reversible droplets under most physiological conditions [21]. Thermodynamic non-equilibrium processes facilitate the constant exchange of substrates and information that allow these condensates to perform important biological functions [22]. The phase transition of these functionally relevant proteins from their native to droplet states are often mediated and stabilized by ATP-dependent factors such as PTM and RNA. RNAs are critical architectural components that can fine-tune biophysical properties such as viscosity and dynamics in the regulation of spatiotemporal distribution of condensates [23,24].

Mutation, mis-regulated RNA processing, and the altered binding of RNA in MLOs that are enriched with RNA and RNA-binding proteins (RBPs) [25] often result in cytotoxicity and the development of neurodegenerative diseases. Aberrant phase separation leading to the pathological amyloid fibrillation of fused in sarcoma (FUS), TAR DNA-binding protein 43 (TDP-43), tau, and α-synuclein (α-Syn} are now associated with neurodegenerative disorders such as amyotrophic lateral sclerosis (ALS), frontotemporal dementia (FTD), Alzheimer’s disease (AD), and Parkinson’s disorder (PD) [26,27,28,29]. The timely dissolution of pathological amyloid fibrils may be dependent on cellular levels of ATP, which has recently been identified as a biological hydrotrope [30]—an amphiphilic molecule that may behave as a surfactant [31] which can reduce tension between solute and solvent, and increase solubility in an energy-independent manner.

## 2. ATP Regulates Biomolecular Condensates

At micromolar concentrations in cells, the hydrolysis of ATP phosphoanhydride bonds provides substantial free energy to fuel chemical processes such as post-translational modifications that may maintain fluid phases or facilitate phase separation by generating supersaturation gradients that can induce droplet segregation [13,15,32,33]. At higher physiological concentrations between 2 and 8 mM, ATP becomes a biological hydrotrope that can solubilize proteins to prevent abnormal aggregation and the formation of pathological amyloid fibrils often associated with neurodegenerative disorders such as Alzheimer’s disease (AD) [30]. Recent extensive all-atom molecular dynamics studies showed that at higher millimolar concentrations (150 mM), ATP prevented the aggregation of amyloid-beta peptide Aβ_16−22_ and disrupted prefibril formations [34], supporting earlier observations of decreased ATP levels in the brain and whole blood of AD transgenic mouse models [35]. Other experimental studies determined that mechanisms such as the suppressed fibrillation of disordered protein by the adenosine moiety of ATP leading to increased protein stability and reduced thermal aggregation may not be typical of hydrotrope-type reactions. Instead, ATP could be viewed as a kosmotropic anion [36] that can increase the solubility of the hydrophobic adenine part [37]; thus, the term “biological aggregation inhibitor” may be more appropriate [38].

Even though ATP is produced mainly in mitochondria, ATP levels in the mitochondrial matrix are significantly lower than those found in the cytoplasm and nucleus [39,40]. Voltage-dependent anion channels (VDACs) located in the mitochondrial outer membrane (MOM) [41] and adenine nucleotide translocators (ANTs) on the inner mitochondrial membranes (IMM) [42,43] facilitate the export of ATP into cytosol where ATP accumulation has been observed to be the highest [44]. The high physiological concentration of ATP in cytoplasm may be used to control the pathological aggregation of macromolecules that coacervate as a result of transient interactions during LLPS in the cytoplasm and nucleus [45,46]. A major hallmark of ALS/FTD is the presence of FUS inclusion in the cytoplasm. FUS are prosurvivor molecules that re-localize from the nucleus to cytoplasm under stress conditions to form reversible, survival-promoting stress granules via LLPS [47,48]. Stress granules contain important ATP-dependent RNA helicases that function as ATPases to hydrolyze ATP during assembly and disassembly [49]. Stress granules could not be formed without the presence of ATP, and the presence of ATP was required to maintain the liquid-like behavior of assembled droplets [32]. A recent in vitro study showed that aggregate disassembly is also an ATP-dependent process.

During metabolic stress such as nutrient deprivation that causes ATP depletion, cells compartmentalize and sequester misfolded proteins into stress granules to protect cellular fitness. Budding yeast subjected to 0.02% glucose starvation showed a 5-fold ATP decline to ~1.1 mM within 10 min, accompanied by a ~4.4-fold increase in median aggregate diameter, whereas the addition of glucose restored ATP levels, quickly reducing aggregate size and abundance back to control values [50]. Mutants with abolished ATP hydrolysis failed to dissolve aggregates even when placed back in 2% glucose solutions after starvation [50]. In the same manner, ATP has been shown to enhance the LLPS of FUS at low concentrations but dissolves FUS aggregates at higher concentrations [51]. Moreover, 8 mM of ATP complexed with Mg^2+^ ions prevented the LLPS of FUS and dissolved previously formed FUS condensates [30]. The presence of ATP facilitates the essential phase transition of FUS into stress granule droplets, yet prevents further transition into irreversible aggregation and the fibrillation of FUS to cause cytotoxicity by binding to the RNA-recognition motif (RRM) domain of FUS, kinetically inhibiting the fibrillization of FUS [52]. Similarly, through binding to arginine-containing domains in TDP-43, ATP altered physicochemical properties to induce LLPS, causing droplet formation at molar ratios as low as 1:100 (protein to ATP); by contrast, increasing ATP concentrations could reduce droplet formation, with TDP-43 droplets completely dissolving at a molar ratio of 1:1000 [53]. Nevertheless, in order to completely dissolve the amyloid-beta peptide Aβ-42 associated with AD, supraphysiological concentrations of ATP in excess of 100 mM were found to be necessary [30].

Tau is the major constituent of fibrillar tangles in AD. Phase-separated tau forms droplets that serve as intermediates toward aggregation [29]. Physiological concentrations of ATP at 0.1–10 mM enhanced the fibrillation of 10 μM tau K18 (equivalent to 10–1000-fold molar ratio) by accelerating aggregation in a concentration-dependent manner [54] through energy-independent binding to tau proteins [55]. It may seem paradoxical that ATP would enhance the formation of amyloids and prions that are associated with diseases. As a matter of fact, prion-like mechanisms are functional biological processes ubiquitously present from bacteria to humans [56]. The nucleation and growth of amyloid fibrils in FUS, TDP-43, tau and α-synuclein are dependent upon intermolecular interactions of intrinsically disordered regions (IDRs) and proteins (IDPs) such as prion-like domains and low-complexity sequence domains [57].

Proteins that undergo LLPS often contain long segments that are intrinsically disordered and lack well-defined three-dimensional structure [58]. The relatively low concentration of hydrophobic amino acids in IDPs enables the rapid exchange between multiple conformations where condensates form without altering the affinity of binding interactions during LLPS [59,60,61]. Although the formation of biomolecular condensates can potentially accelerate amyloid aggregation, condensates can also inhibit fibril formation by the sequestration of aggregation-prone, prion-like IDPs. Biomolecular condensates derived from proteins associated with the formation of processing bodies (P-bodies) prevented aberrant amyloid aggregation despite local increase in concentration of aggregate-prone proteins [62].

P-bodies are conserved eukaryotic cytoplasmic ribonucleoprotein (RNP) membraneless organelles that regulate protein homeostasis in non-stressed cells through LLPS involving messenger RNAs (mRNAs) and low-complexity sequence domains [63,64,65,66]. P-bodies respond to cellular stress, especially DNA replication stress, by increasing their sizes and numbers [67,68]. The disassembly of P-bodies in yeast is an ATP-dependent process involving ATP hydrolysis by DEAD-box ATPases [69]. The formation of P-bodies is dependent upon RNA and non-translating mRNAs [67]; therefore, it is not surprising to find P-bodies located very close to endoplasmic reticulum (ER) membranes [70] enriched with membrane-associated mRNAs [71,72,73]. Native tau proteins are stable, highly soluble, and resistant to aggregation. When these intrinsically disordered proteins interact with anionic lipid monolayers in plasma membranes, they will undergo LLPS, transitioning from a disordered monomeric state to a pathogenic fibrillar state [74,75]. Phase-separated tau easily aggregates to form highly ordered β-sheets often associated with neurodegeneration [76,77]. The dynamic crosstalk between membranes and membraneless organelles highlights important features critical to the functions and maintenance of biomolecular condensates in health and disease [78,79].

## 3. The Interdependence between Membranes and Membraneless Organelles

Efficient cellular compartmentalization with or without membranes is indispensable for organic and prebiotic inorganic life [80,81]. There exists a tight interdependence between membranes and MLOs. Since its first discovery in the 1830s, MLOs have been found not only in the nucleus and cytoplasm, but on the membranes of almost all eukaryotic cells [82]. In eukaryotes, lipid bilayer plasma membranes form dynamic trafficking networks with the elaborate endomembrane systems comprising membrane-bound organelles such as the endoplasmic reticulum, Golgi apparatus, endosomes and lysosomes [80,83]. However, exact mechanisms that regulate signaling events and control cargo protein movements within this complex membrane network are not fully understood [84,85]. MLOs formed at membrane surfaces may regulate receptor/transmembrane protein signaling by increasing protein binding affinity and modulating local environments [86]. Recent discoveries revealed that the cluster stoichiometry of condensates formed at plasma membranes could fine-tune signaling proteins such as Ras by increasing dwell time to facilitate kinetic proofreading receptor-mediated activation [87,88]. Conversely, membranes are major regulatory platforms for LLPS due to their ability to concentrate and change protein thresholds during phase separation [79]. Membrane surfaces acted as catalytic sites where alterations in membrane fluidity and lipid composition increased Aβ-42 peptide aggregation and facilitated the binding and internalization of pathogenic amyloid fibrils [89]. β-amyloid peptide (Aβ) featured in AD are derived from amyloid precursor proteins (APPs) where APP cleavage by β-secretase or α-secretase will initiate the amyloidogenic or nonamyloidogenic processing of APP, respectively [90]. Conversely, alterations in membrane fluidity from lipid composition fluctuations such as the reduction in cholesterol and increased membrane fluidity induced nonamyloidogenic APP cleavage by α-secretase [91]. The two distinct pools of APP cleaved by α- and β-secretase that were discovered to exist outside and inside of lipid rafts, respectively [90], may be the result of interactions between lipid rafts and biomolecular condensates.

### 3.1. Lipid Rafts and Biomolecular Condensates in Health and Disease

Since K. Simons first presented the concept of lipid rafts in 1997 as clusterings of sphingolipids and cholesterol-forming mobile microdomain platforms responsible for signal transduction and protein transport [92], these phase-separated regions in lipid bilayers have been associated with relevant biological functions, including signal transduction [93], trafficking, and the sorting of proteins and lipids [94,95]. Lipid raft signaling is implicated in the pathogenesis of numerous diseases [96], including neurodegenerative disorders [90,97], cardiovascular disease [98], prion disease [99,100], systemic lupus erythematosus [101], viral replication [102], and tumorigenesis [103]. Numerous cancer-related proteins that may be involved in migration, invasion, and metastasis are localized in lipid rafts, understood to be signaling hubs for these proteins [104,105,106]. Gene transcription has been shown to be regulated by biomolecular condensates [107,108,109]; therefore, the recent association of mutations in cancer-related genes with aberrant phase-separated biomolecular condensates [10,110] emphasizes essential relationships between membranes, lipid rafts, and ATP that may not be fully elucidated.

Membrane surfaces offer many advantages in the formation of condensates such as increased pi–pi and cation–pi interactions imposed by geometrical constraints on a two-dimensional flat surface [111], which can reduce the requirements for critical the concentration of molecules necessary for phase separation [112] and enhance biochemical reactions that may take place within biomolecular condensates [113]. Many MLOs form near lipid membrane surfaces because they may rely on lipid-anchored proteins, including H-ras [87,114] which are often found in lipid rafts, for spontaneous thermodynamic phase separation into distinct domains [5,115]. Lipid rafts may enhance phase separation; lipid-driven phase separation within lipid rafts has been demonstrated to dynamically interact with the phase separation of membrane-anchored proteins, resulting in combined effects that change the final phase separation outcome of both systems while enhancing protein-driven phase separation [116]. Indeed, the formation of linker for activation of T cells (LAT) condensates on membrane surfaces induced lipid phase separation into distinct liquid-ordered (L_o_) lipid raft domains [117]. To remain in functional states, bimolecular condensates may require energy to support the continuous active restructuring and rearrangement of molecular components. Insufficient or the depletion of ATP can directly impact the physical and functional properties of biomolecular condensates [32,33,79,118]. 

### 3.2. Non-Mitochondrial Dimerized ATP Synthase and ATPase Are Localized in High-Curvature Lipid Rafts/Caveolae

First isolated in 1960 [119,120], F_1_F_0_ ATP synthases are found localized in the inner membrane invaginations of mitochondria [121]. Eukaryotes and prokaryotes use four major types of ATPases localized in cell membranes to release energy during hydrolysis of ATP for the maintenance of critical transmembrane ionic electrochemical potential differences [122]. In the ubiquitous intracellular powerhouses of eukaryotes, F_1_F_0_ ATP synthase is complex V of the electron transport chain responsible for chemiosmotic oxidative phosphorylation (OXPHOS) that couples ATP synthesis to the inner membrane proton gradient [123,124,125]. Of the four types of ATPases—F_1_F_0_, P [126], V [127], and ABC [128]—only F_1_F_0_ ATPase can reverse the rotation direction of its γ-subunit to function as ATP synthase, binding inorganic phosphate (Pi) to adenosine diphosphate (ADP) to form ATP [129,130], whereas P-type ATPases are mostly found on plasma membranes [126], and V-type ATPases are located on plasma membranes as well as the membranes of intracellular organelles including endosomes, lysosomes, and the Golgi network [127,131]. Once believed to be exclusive to inner membranes of mitochondria, since 1994, non-mitochondrial extracellular F_1_F_0_ ATP synthases have been discovered on plasma membrane surfaces of a variety of cell types, including numerous tumor cell lines [132,133], endothelial cells [134], human umbilical vein endothelial cells [135], HepG2 [136,137] and hepatocytes [138], HaCaT keratinocytes [139], and even in neurofibrillary tangles associated with AD [140].

Although the F_0_ domain of both bacterial and eukaryotic ATP synthase is embedded within the plasma membrane, the hydrophilic, water-soluble catalytic F_1_ domain of eukaryotes is oriented towards extracellular space, whereas that of bacteria is directed inwards toward the cytoplasm [130,141]. Both bacteria and eukaryotes use membrane-bound, non-mitochondrial ATPase/ATP synthase to calibrate the homeostasis of intracellular pH [141,142,143,144,145,146]. Intracellular pH is an important regulator of biomolecular condensates because macromolecules including RNA and proteins undergo LLPS as adaptive, reversible, quick responses to subtle environmental stimuli that may include changes in pH, salt concentration, and temperature [3,147,148,149]. The localization of ATP synthase in caveolae, which are uniform, bulb-shaped, specialized lipid raft invaginations in plasma membranes, may confer protection to the proton gradient required for the transfer of protons into extracellular space to maintain intracellular pH and to power F_1_F_0_ rotors during ATP synthesis [141,150].

For the first time, in 2004, ATP synthase alpha and beta were discovered to be expressed in lipid rafts isolated from rat HepG2 hepatocytes by immunofluorescence [136], and a functionally active F_1_F_0_ ATP synthase on isolated rat hepatocytes plasma membrane was independently confirmed a few years later [151]. In the same year (2004), significant levels of ATP synthase complex capable of generating extracellular ATP and regulating plasma membrane proton gradient were found in lipid rafts of human adipocytes [152]. P-type Ca^2+^-ATPases [153] such as plasma membrane Ca^2+^ ATPase (PMCA) were found to be exclusively localized to cholesterol/sphingomyelin-rich lipid raft domains of caveolae in pig cerebellum synaptic plasma membranes [154], and sarco/endoplasmic reticulum Ca^2+^ ATPases (SERCA) were similarly identified in caveolae/lipid rafts in human uterine cells [155], rat hepatocytes [156], and human Müller glial cells of the retina [157]. Capable of dynamic responses to stimuli through rapid formation and dissipation [158], caveolae are subsets of lipid rafts enriched in glycosphingolipids, cholesterol, sphingomyelin, and lipid-anchored membrane proteins [159,160,161]. Cholesterol is a high-curvature lipid that creates spontaneous negative curvature in lipid bilayers [162,163,164], and naturally accumulates in high-curvature regions of lipid domains such as caveolae invaginations and lipid rafts [165]. In order to maintain curvature and their unique invaginations, caveolae recruit caveolins to bind and increase cholesterol concentration in a 1:1 ratio [166,167]. The fact that most non-mitochondrial ATP synthases and ATPases are highly localized in caveolae and lipid raft domains [136,152] is reminiscent of ATP synthase dimers that exclusively localize in high-curvature cristae invaginations of inner mitochondrial membranes (IMMs).

#### 3.2.1. Dimerized ATP Synthase/ATPase Require High-Curvature Lipid Domains

The ATP synthases of mammalian mitochondria are usually arranged in rows of dimeric complexes of two identical monomers located at the highly curved apex of deep IMM invaginations known as cristae [168]. Dimerized ATP synthases are seven times more active than monomers [169]. Dimerization of ATP synthase may be a major determinant in cristae formation [170], because extreme cristae membrane curvature is shaped by the self-assembly of ATP monomers into dimerized rows [171]. Inability to form dimers resulted in reduced or deformed cristae invaginations [172] that impacted ATP production from decreased OXPHOS activity as a result of defective cristae morphology [173,174]. Experimentally purified ATP synthase reconstituted with membrane lipids revealed that dimerized rows of ATP synthases were formed only on curved surfaces and not on flat membrane areas [175]. Extracellular F_1_F_0_ ATP synthases have been observed to translocate from mitochondria to lipid raft domains of various cell types, including plasma membranes of gonadotropes [176], and the sarcolemma of muscle fibers [177].

#### 3.2.2. Translocation of ATP Dimers to Lipid Rafts Are Cellular Responses to Stress and Stimuli

Biomolecular condensates adapt to changing endogenous or exogenous conditions [3] by continuously fine-tuning biochemical reactions, enriching or excluding biomolecules from their environment [7]. The rapid translocation of mitochondrial ATP synthase to lipid rafts may be integral to these adaptive responses because ATP functions not only as a biological hydrotrope [30,178], increasing the solubility of positively charged, intrinsically disordered proteins [179], but may act as a universal and specific regulator of intrinsically disordered regions (IDRs) capable of altering physicochemical properties, conformation dynamics, assembly, and aggregation [45], in addition to providing phosphates as an energy source to fuel post-translational modifications that regulate the fluctuation of biomolecule phase separation during condensate formation [79,178]. 

LLPS is further regulated by lipid raft membrane-anchored proteins that support the continuous restructuring and rearrangement of molecular components in condensates [116]. Cell surfaces from six different cell lines, including human umbilical vein endothelial cells (HUVECs), human hepatocellular liver carcinoma cells (HepG2), hepatic cells (L-02), human highly metastatic lung cancer cells (95-D), human lung cancer cells (A549), and human embryonic kidney cells (293), revealed that there were significant ATP synthase translocations from mitochondria and an upregulation of catalytic activities under tumor-like acidic and hypoxic conditions compared to normal conditions [180]. Upon edelfosine-induced membrane permeability resulting in the depolarization and disruption of IMM proton gradients, mitochondrial F_1_F_0_ ATP synthases in various human cancer cell lines translocated to cell surface lipid rafts or to lipid raft domains present in mitochondria [181]. The presence of redox signaling [182,183] and cancer-related [104,105] proteins in lipid rafts/caveolae further emphasizes the importance of lipid raft domains in health and disease [96,184]. Failure to maintain nanoscopic lipid raft domains with appropriate line tension and membrane elasticity [185] to functionally host dimerized ATPase [186], ATP synthase [175] may contribute to aberrant phase separation, resulting in pathogenic protein aggregates in neurodegeneration [11] and cancer [10,12].

### 3.3. Physiological Nanoscopic Lipid Raft Domains Are Stabilized by Intrinsic Negative Membrane Curvature and Reduced Line Tension

Lipid bilayers in cell membranes are composed of hundreds of different lipid species with a propensity to segregate laterally into subcompartmentalized raft domains [187,188]. Found in plasma membranes, intracellular membranes, and extracellular vesicles, lipid rafts are dynamic, nanoscopic (10–200 nm), transient, mobile, liquid-ordered (Lo) domains formed as a result of thermodynamically driven LLPS [189,190,191,192,193]. Compared to non-raft domains, the lower-fluidity transient lipid rafts serve as signaling hotspots that respond to external stimuli by modulating their composition and size, and increasing or lowering the concentration of signal transduction proteins [93,194,195]. When formed under pathological inflammatory conditions, lipid rafts become enlarged inflammarafts (i-rafts), signaling platforms that contain activated receptors and adaptor molecules associated with inflammatory cellular processes in diseased states [196,197,198]. Enlarged lipid rafts often serve as scaffolding platforms that aggregate pro-inflammatory NLRP3 inflammasome [196] and cluster pro-apoptotic signaling molecules (CASMER) commonly found in many types of cancer [104,199]. The important roles played by lipid rafts in neurological disorders such as Alzheimer’s disease have long been appreciated [200,201,202,203]. The destabilization and changes in the lipid composition of rafts due to elevations of lipid peroxidation from natural aging [204] offer additional insight into the important relationship between membranes and MLOs in the molecular pathophysiology of neurodegenerative disorders. 

Line tension, or the energy required to create boundaries between lipid raft domains and the surrounding membranes, is one of the key drivers that can determine the size, form, and shape of physiological nanoscopic lipid compartments [185]. The hydrophobic mismatch between lipids in raft domains increases the energy and line tension required to maintain rafts as separate compartments [205]; therefore, a reduction in line tension will minimize the free energy between ordered and disordered liquid phases and contribute to the more efficient formation of physiological nanoscopic rafts [188]. Interestingly, the intrinsic, spontaneous curvature of membranes has been demonstrated to be able to reduce line tension [185,206]. Nanoscopic lipid raft domains are generated and stabilized by coupling lipid monolayers with different spontaneous curvatures in liquid-ordered (L_o_) and liquid-disordered (L_d_) phases to induce elastic interactions by reducing line tension between L_o_ and L_d_ phases [207]; in addition, lowering line tension through enrichment with high-curvature lipids such as cholesterol successfully induced the transition from macroscopic to nanoscopic L_o_ phase lipid raft domains [208]. Furthermore, the in vitro loading of cholesterol enhanced both the abundance of cholesterol in the caveolae/lipid rafts of human umbilical vein endothelial cells (HUVECs) and translocation of the ATP synthase beta chain responsible for catalysis in F_1_ domains to cell surfaces while significantly doubling the degree of extracellular ATP production within 30 min of exposure [209,210].

The ability of ATP synthase/ATPase to form dimerized rows on the IMM of mitochondria and other membrane surfaces may be highly dependent upon membrane lipid composition [211] and curvature [175]. Uncontrolled, excess oxidative stress can cause lipid peroxidation [212] which induces pathological changes to membrane lipid composition, including alterations of cardiolipin in IMMs [211,213], as well as changes in membrane curvature that prevent optimal dimerization and the subsequent functioning of ATP synthase/ATPase [214,215]. Insufficient or depletion of ATP can directly impact the physical and functional properties of biomolecular condensates [32,33,79,118]. ATP is not only a biological hydrotrope capable of inhibiting protein LLPS and aggregation at high mM concentrations; it has recently been observed to act as a universal and specific regulator of IDRs, altering their physicochemical properties, conformation dynamics, assembly, and aggregation [45]. Furthermore, ATP has been documented to associate with phospholipid bilayers, forming aggregates at high mM concentrations in the aqueous phase. In fact, the endogenous heterogeneity of lipid membranes was seen to selectively enhance the diffusion restriction of ATP in the cytosol [216].

### 3.4. Oxidative Stress Alters Lipid Molecular Structures in Rafts and Membranes, Resulting in the Accumulation of Pathological MLOs

Inability to neutralize excess reactive oxygen species (ROS) accumulated as products of normal cellular functions results in a state of imbalance often referred to as oxidative stress [217]. Oxidation of lipids in membranes disrupts functionality by inducing changes in lipid molecular structure that leads to diminished negative intrinsic membrane curvature, lowered membrane fluidity, and increased membrane permeability [214,218,219,220]. Phase separation of biomolecular condensates such as FUS takes place in the cytoplasm. The presence of high levels of ATP in cytoplasm can ensure proper dissolution of FUS aggregates [51,52]. Even though mitochondria are major ATP-producing organelles in eukaryotes, ATP concentration in mitochondria is maintained at significantly lower levels than that of cytoplasm [40] by voltage-dependent anion channels (VDACs) located in the mitochondrial outer membrane (MOM) [41] and adenine nucleotide translocators (ANTs) on the IMM [43] that transport ATP from mitochondria into cytoplasm. Therefore, mitochondrial ATP production exerts a direct influence on the formation and dissolution of MLOs in cytoplasm. Importantly, the amount of ATP produced in mitochondria is, in large part, determined by cristae morphology [221].

Cristae are dynamic, independent, bioenergetic IMM invaginations capable of remodeling in seconds to organize respiratory chain supercomplex assembly and ATP synthase for efficient ATP production [221,222]. Mitochondrial membrane lipid composition may contain up to 24–25% of cardiolipin (CL) [223,224,225]—an anionic, high-curvature, four-acyl chain lipid with a unique cone shape that can stabilize negative membrane curvatures in cristae and increase bending elasticity of the IMM [226,227,228,229,230,231]. Embedded in the IMM cristae, the F_0_ motor of the ATP synthase controls proton flux that powers the rotation of the F_1_ subunit protruding into the mitochondrial matrix, driving the synthesis of ATP [232]. CL is required for the proper docking and insertion of OXPHOS proteins into the IMM, as well as the formation and maintenance of structural integrity of the mitochondrial respiratory chain supercomplexes [233,234]. This is probably why CL binds to the F_1_F_0_ ATP synthase with higher affinity than all other mitochondrial phospholipids [235]. ATP synthesis could be significantly enhanced when proton translocation is increased by the non-bilayer structures at the apex of IMM cristae formed during CL interactions with the F_0_ section of ATP synthase [236], whereas CL deficiency can result in compromised mitochondrial energetic and coupling efficiency in skeletal muscles [237]. Mitochondrial bioenergetics are heavily dependent upon optimal CL lipid composition, content, and structure [238]; therefore, mitochondrial dysfunction as a result of CL peroxidation and depletion is associated with numerous pathophysiological conditions [239], including myocardial ischemia [240], nonalcoholic fatty liver disease [241], thyroid dysfunctions [242], diabetes, obesity and other metabolic diseases [243,244], cancer [245], as well as a wide range of neurological disorders including Alzheimer’s disease [246], Parkinson’s disease [247,248], amyotrophic lateral sclerosis [249], Barth syndrome [250,251], and traumatic brain injury [252,253]. The highly unsaturated phospholipids in CL are extremely sensitive to ROS attack. CL oxidation products in animal models may be used as effective biomarkers for oxidative stress in mitochondria [254,255]. Alterations in lipid composition and molecular structure, as well as membrane curvature and line tension as a result of ROS attacks, often initiate signaling events that recruit MLOs to membrane sites, whereas pathological amyloidogenic MLO aggregates at membranes, in turn, alter membrane structures [256,257].

### 3.5. ROS-Externalized Cardiolipin Facilitates the Accumulation of Amyloid/Prionoid Aggregates and Activates Autophagic and Inflammatory Signaling

Cardiolipin (CL) is a mitochondria signature lipid distinctly attracted to membrane lipid domains with strong negative curvatures, such as the apex of IMM cristae [226,228]. CL is often externalized to the outer mitochondrial membrane (OMM) upon mitochondrial distress from ROS attacks [258,259], whereas oxidized CL in OMM initiates apoptotic signaling processes [260] that can lead to opening of the mitochondrial permeability transition pore (mPTP) and the release of cytochrome c (Cyt c) [261,262]. Externalized CL, whether oxidized or not, becomes an essential signaling platform that binds and interacts with important mitophagic, autophagic, and inflammatory enzymes [259,263], including Beclin 1 [264], tBid, Bax [262,265], caspase-8 [266], and the NLR pyrin domain containing 3 (NLRP3) inflammasomes [267]. A major source of extremely inflammatory cytokines IL-1β and IL-18 [268], NLRP3 inflammasome is a phase-separated supramolecular complex that mediates immune responses upon the detection of cellular stress and dysfunction [269,270,271]. The activation of the NLRP3 inflammasome in macrophages is induced by oxidized phospholipids [272], whereas the docking of externalized CL to NLRP3 inflammasome primes its assembly and subsequent activation in mitochondria [267] as well as mitochondria-associated membranes (MAMs), a region comprising highly specialized proteins which is tethered to the endoplasmic reticulum (ER) [273,274]. ER stress and MAM dysfunction are increasingly associated with the aggregation of misfolded proteins as a result of aberrant phase separation [275,276,277]. The conversion of the phase-separated presynaptic neuronal protein α-syn from a physiological liquid-like droplet state into the pathological amyloid hydrogel aggregated state may also be facilitated by binding with externalized CL at OMM, ultimately disrupting mitochondrial membrane integrity and enhancing neurotoxicity [278,279,280]. Neurodegenerative disorders such as AD and PD have been associated with aberrant CL content, structure, and localization [281].

α-syn demonstrates a high affinity for mitochondrial membranes, interacting in close proximity with mitochondrial OXPHOS proteins, including lipid raft-like domains at MAMs that are high in phospholipids [282,283]. Native, unfolded, monomeric α-syn improves ATP synthase efficiency and increases ATP levels [284,285], whereas the pathological aggregation of α-syn can generate ROS to cause lipid peroxidation and the oxidation of ATP synthase beta subunits, inhibiting mitochondrial respiration [286], opening mPTP, and resulting in apoptosis [287]. CL has been observed to enhance the formation of ion-permeable pore structures with channel-like properties by α-syn oligomers in lipid membranes [288]. MAMs, IMM, and OMM, with their lipid raft-like domains enriched with CL, easily form pores large enough to allow the transit of water and other small molecules that could cause mitochondrial swelling and Cyt c release [288,289,290]. Intriguingly, most ion channels preferentially reside in membrane raft-like microdomains [291].

Physiological lipid rafts function optimally at nanoscopic sizes [292,293,294]. ROS that attack anionic lipid headgroups at membrane interface [295] can cause lipid peroxidation cascades, creating products that alter raft properties, and increasing line tension [206] to grow nanometer-scale rafts into enlarged, micron-sized inflammarafts [196,197,296] that carry pro-inflammatory signaling molecules [104,198,199]. Melatonin, known for its modulatory effects on various ion channels [297,298,299], has recently been observed to directly inhibit cryopreservation-induced mPTP opening, increasing ATP production, counteracting OXPHOS inhibition, as well as upregulating glycolysis [300]. The fact that oxidized CL, whether exogenously added [261] or endogenously induced [301], causes mPTP opening in mitochondria, further accentuates the necessity for the timely resolution of oxidative stress by appropriate antioxidants.

### 3.6. Melatonin Inhibits Cardiolipin Peroxidation to Prevent the Aggregation of Pathological MLOs at Membranes

**Figure 1:**
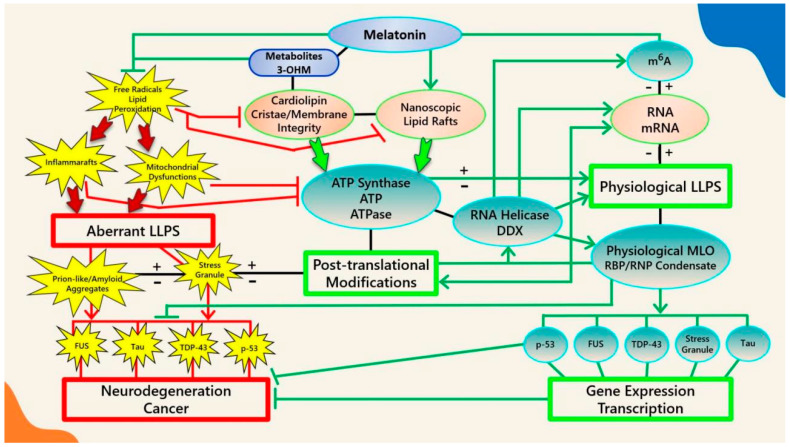
Schematic illustrating the regulation of biomolecular condensates by melatonin represented through observations reported in antioxidant protection against lipid peroxidation to maintain membrane/lipid raft composition/stability that serves to maintain adequate ATP levels in all cellular compartments to fuel, support, and regulate post-translational/m^6^A modifications that may fine-tune RNA dynamics in the assembly and disassembly of MLOs to prevent pathological aggregations in neurodegenerative disorders. LLPS: liquid–liquid phase separation; DDX: Dead-box RNA helicase; m6A: N^6^-methyladenosine; MLO: membraneless organelle; RBP: RNA-binding protein; RNP: ribonucleoprotein; PTM: post-translational modification (See Abbreviations for additional acronyms).

**Figure 2:**
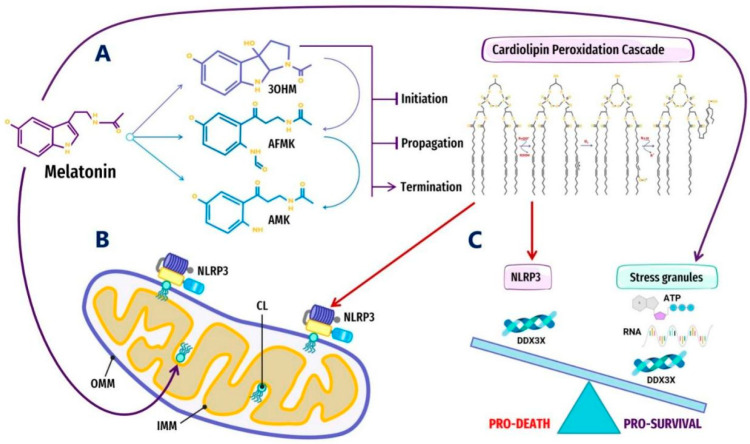
Overview of melatonin regulation of NLRP3 inflammasome (NLRP3) formation, assembly and activation: (**A**) Summary of melatonin and metabolite antioxidant cascade inhibiting the initiation and propagation of cardiolipin (CL) peroxidation, effectively terminating the CL peroxidation cascade; (**B**) Oxidized CL is externalized from the cristae/inner mitochondrial membrane (IMM) to the outer mitochondrial membrane (OMM) where it docks and primes NLRP3 inflammasome assembly prior to activation in mitochondria; (**C**) DDX3X, an ATP-dependent DEAD-box RNA helicase, is the mediator that selects the formation of “Pro-Survival” stress granules or the transition of the NLRP3 inflammasome into “Pro-Death”, stable, prionoid-like complexes. The successful formation of stress granules is also dependent upon the availability of ATP and RNA, both of which may be regulated by melatonin (See Abbreviations for additional acronyms).

Melatonin is a potent antioxidant that has been shown to inhibit CL peroxidation in mitochondria, preventing mPTP opening and Cyt c release [301] by inhibiting peroxidation cascades initiated by specific ROS that accumulate in lipid headgroups at membrane–water interfaces [295] (Figure 1). The suppression of oxidative stress and lipid peroxidation may halt the externalization or oxidation of CL, effectively preventing potential pathological interactions with MLOs such as α-syn and the NLRP3 inflammasome. The interaction between pathological α-syn oligomers and externalized CL can result in increased ROS, lipid peroxidation, and mitochondrial dysfunction; therefore, it is not surprising that melatonin has been demonstrated to block α-syn fibril formation and oligomerization, decreasing cytotoxicity in primary neuronal cells [302], as well as rescuing impaired mitochondrial respiration induced by α-syn in *Saccharomyces cerevisiae* under ROS attack [303]. The NLRP3 inflammasome must be primed by externalized CL upon ROS stimulation before activation [258,267,273]. The regulation of the next phase where the NLRP3 inflammasome transitions into stable, prionoid-like complexes is mediated by DDX3X, one of the ATP-bound forms of DEAD-box RNA helicases responsible for the scaffolding of prionoid, self-oligomerizing specks known as apoptosis-associated speck-like protein containing a C-terminal caspase recruitment domain (ASC) which cannot be easily disassembled once they are formed [304,305,306] (Figure 2).

ATP-dependent DEAD-box RNA helicases (DDXs) are ATPases that regulate RNA-containing phase-separated organelles in prokaryotes and eukaryotes [307,308]. DDXs promote phase separation in their ATP-bound form, but can also release RNA and induce compartment turnover using ATP hydrolysis. Inhibition of DDX ATPase activity can disrupt the disassembly of physiological MLOs such as P-bodies and stress granules [69,309] (Figure 1). It is presently unknown what prompts DDX3X to select the aggregation of pro-survival stress granules over pro-death NLRP3 inflammasomes or vice versa [304,310]. It would not be unreasonable to assume that an excessive oxidative local environment with pathological i-rafts in membranes could exert a decisive influence over the selection process (Figure 2).

The activation of the NLRP3 inflammasome is now associated with major neurodegenerative disorders such as AD, PD and ALS, where positive correlations have been found to exist between NLRP3 levels and abnormal protein aggregations such as Aβ and α-Syn, whereas the inhibition of the NLRP3 pathway attenuates pathological protein aggregations [311]. Melatonin inhibited NLRP3 inflammasome activation and reduced the aggregation of ASC specks in the mice hippocampus with major depressive disorder induced by inflammatory liposaccharides [312]; melatonin also inhibited the formation of hypoxia-induced inflammasome protein complexes and reduced the aggregation of ASC specks in macrophages of Sugen/hypoxia pulmonary arterial hypertension (PAH) mouse models [313]. Melatonin attenuated the progression of intervertebral disc degeneration in vitro and in vivo by reducing mitochondrial ROS products to inhibit NLRP3 inflammasome priming and activation, effectively terminating pro-inflammatory cytokine expression [314]. The ability of melatonin to prevent the opening of mPTP and release of Cyt c [301], inhibit NLRP3 inflammasome priming, activation, and ASC speck aggregation [312,313], block α-syn fibrillation [302], and improve mitochondrial respiration [303] could be directly related to its ability to stabilize nanoscopic lipid raft domains and suppress lipid peroxidation, which can alter the composition and molecular structures of lipid rafts.

### 3.7. Melatonin Regulates Membrane Lipid Dynamics and Composition via Phase Separation

Nanoscopic transient lipid raft domains in biological membranes are formed by phase separation in response to external stimuli [92,93,188]. Even though cells may alter lipid constituents to control the composition and size of lipid rafts [315], the propagation of molecular stress, lipid raft rattling dynamics and relaxation are some of the basic mechanisms underlying phase separation on the molecular level [195]. The presence of hydrophobic molecules such as melatonin can modulate viscoelastic dynamics through the accumulation and propagation of stress in lipid–lipid interactions [195,316]. Adding melatonin to membrane models led to a breakdown of out-of-phase membrane displacement patterns and the disruption of the vibrational landing platform of lipid biomolecules at the water–membrane interface, effectively slowing the permeation of ROS and other small molecules [195,317].

In 2005, melatonin was first observed to induce phase-separation in DPPC lipid bilayers [318]; recently, melatonin has been observed to modify lipid hydrocarbon chain order to promote phase separation in ternary membrane models [319]. Due to a preference to localize at membrane interfaces [320], melatonin can form strong hydrogen bonds with membrane lipid anionic headgroups that could significantly modulate lipid acyl chain flexibility and lipid dynamics [318]. Melatonin is able to directly interact with cholesterol [321] and displaced cholesterol due to competitive binding to lipid molecules, increasing disorder in the L_d_ phase to drive cholesterol into the ordered L_o_ phase [319]. These subtle changes in lipid nanodomains can profoundly affect amyloid processing at membrane sites. Aβ_1–40_ and Aβ_1–42_ peptides are known to interact strongly with negatively charged lipids by binding to anionic, negatively charged membranes [322,323,324,325,326]. Increasing cholesterol content lowered the surface charge of lipid membranes in saline solution from positive to negative [327]. Although cholesterol is an indispensable constituent of lipid rafts [92,162], its electrostatic properties altered interactions of charged or polar biomolecules on lipid membrane surfaces and attracted the targeted binding of Aβ deposits at lipid membranes [328,329,330,331].

In animal and in vitro studies, melatonin was able to prevent or ameliorate tau and Aβ pathology in AD [332,333,334,335] and inhibit Aβ production and assembly while enhancing non-amyloidogenic APP processing [336]. As early as 1998, melatonin was documented to inhibit amyloid fibrillation through modifications of Aβ peptide secondary structures. It was hypothesized that the observed changes could have been due to the unique structural characteristics as well as antioxidant properties of melatonin [337]. As a result of deficient melatonin from natural aging, Aβ_25–35_ peptides embedded in hydrocarbon cores of anionic lipid bilayers may further displace cholesterol molecules to increase oligomerization or fibrillation [338], but the addition of 30 mol% melatonin to anionic membranes strikingly reduced membrane-embedded Aβ peptides [338]. Melatonin behavior in membrane systems was affected by the competitive binding dynamics between melatonin and cholesterol to membrane phospholipids via hydrogen bonds. The presence of cholesterol could also change melatonin configuration from folded to extended, whereas increasing cholesterol levels to 50% drove melatonin from the membrane interface to become fully solvated by lipid headgroups or bulk water [339]. On the other hand, a single, intraperitoneal, pharmacological dose of melatonin at 100 mg/kg strengthened hydrogen bonding in the polar zone and increased disordering in the non-polar zone of phospholipids in rat brain membranes [340].

Local variations in melatonin concentration also affected the re-ordering of lipids in membranes. At 0.5 mol% concentration, melatonin was documented to penetrate lipid bilayers to form fluid domains that enriched lipid membranes where melatonin molecules aligned parallel to phospholipid tails with the electron-dense regions slightly below hydrophilic headgroups; however, at 30 mol% concentration, melatonin molecules aligned parallel to the lipid bilayer, close to the headgroup regions where one melatonin molecule was associated with two lipid molecules to form an ordered, uniform, lateral membrane structure distributed evenly throughout the membrane model [341]. Variations in local concentration and conformational changes in melatonin molecules can directly impact the lipid phase transition, line tension, size, health, and functions of lipid rafts. 

### 3.8. Melatonin Increases Membrane Fluidity and Reduces Line Tension to Stabilize and Maintain Nanoscopic Lipid Raft Domains

Membrane fluidity reveals the degree of molecular disorder and motion within lipids in membrane bilayers [342]. There are hundreds of different lipid species in lipid bilayers that have a high propensity to segregate laterally into subcompartmentalized lipid raft domains [187]. Oxidative stress can increase membrane rigidity, altering lipid raft formation rates as a response to cellular stress [343,344]. Oxidation of lipids in membranes can also alter molecular structures by creating amphiphilic subpopulations leading to significant changes in the phase behavior of lipid membranes that can affect the integrity and structure of membranes [214]. When under ROS attack, cells form cubic lipid structures in the smooth endoplasmic reticulum and IMM [214,345,346]. 

It is believed that lipid rafts function optimally as nanodomains [114,293], whereas rafts that are enlarged under inflammatory conditions assemble pathological MLOs associated with cellular processes in diseased states [104,196,197,198,199]. Essentially, lipid peroxidation alters the organization, assembly, and structure of membrane lipids [256,347,348], where lipid peroxides often induce nanometer-scale rafts to grow to micron sizes, accompanied by increased line tension in the order of several piconewtons [206,218,296]. Lipid peroxidation also prevents the formation of lipid rafts at room temperature by enhancing phase separation that favors significant increases in the fraction of the non-raft L_d_ phase [349]. Interestingly, melatonin was observed to stabilize lipid L_o_–L_d_ phase separation over a range of temperatures and domain sizes, effectively preventing the formation of a non-raft L_d_ phase, possibly by reducing line tension or acting as a surfactant at L_o_–L_d_ interfaces [350]. ATP is possibly a surfactant [30,31] capable of reducing the interfacial free energy penalty during the formation of smaller-sized multiple coexisting MLOs, whereas larger droplets may form as a result of lower surfactant ratios [351]. Whether melatonin can also act as a surfactant [350] to induce the formation of small, multiple coexisting droplets may require further validation while increasing evidence is being reported [352]. Nonetheless, by stabilizing and maintaining optimal nanoscopic lipid domains, melatonin is perfectly capable of preserving the high level of cytosolic ATP concentration requisite for proper biomolecular condensate formation and dissolution through its features as a potent antioxidant.

During lipid peroxidation events, oxidized moieties were found to mainly reside close to the lipid headgroups forming hydrogen bonds with water. These oxidized lipids can perturb membrane bilayer structures and modify membrane properties, including decreasing the membrane fluidity [318,353,354,355]. The preferential location of melatonin in bilayer lipid headgroups allows dynamic interactions that lead to reductions in bilayer thickness and increased bilayer fluidity [338,341,356]. Eukaryotes and prokaryotes use ATPases localized in cell membranes and lipid raft domains to produce and release ATP energy [122,127,136,152]; therefore, increased ATPase activities from enhanced membrane fluidity [357,358] can impact how ATP interacts with phospholipids in bilayers [216] and modulate the LLPS of MLOs formed at membrane surfaces [45]. Moreover, lipid peroxidation is believed to be associated with the reduction in mitochondrial membrane fluidity during aging in animals [359]. Membranes themselves can affect local protein concentrations [360] where high-curvature lipids that form rafts may attract specific proteins that form aggregates to further enhance membrane curvature [361,362,363,364]. Increasingly, neurodegenerative diseases such as AD are viewed as membrane disorders [203]. The size of MLOs that aggregate at membrane surfaces can be tuned through PTMs such as phosphorylation, which is ATP-dependent [365]. The amount of ATP available at membrane surfaces and cytosol drives the formation, tuning, and dissolution of MLOs, and is regulated by oxidative-stress-sensitive ion channels that reside in lipid rafts (Figure 1).

### 3.9. Melatonin Maintains a High Cytosolic ATP:ADP Ratio through the Optimization of VDAC-CYB5R3 Redox Complexes in Lipid Rafts

Lipid rafts are phase-separated regions in lipid bilayers responsible for important biological functions including signal transduction [92,93] as well as the trafficking and sorting of proteins and lipids [94,95]. The fact that lipid rafts are also important redox signaling platforms that assemble, recruit, and activate redox regulatory multiprotein complex NADPH oxidase [182,366], and host the quintessential plasma membrane redox enzyme complex VDAC-CYB5R3 [367,368], emphasizes the relevance of melatonin as an antioxidant in the protection and stabilization of lipid raft domains.

Present in all eukaryotes [369], CYB5R3 encodes for a NADH-cytochrome b5 reductase 3 flavoprotein that is engaged in the one-electron transfer from NADH to cytochrome b5 or plasma membrane coenzyme Q, producing NAD^+^ as a result [370,371]. The soluble isoform of CYB5R3 is exclusive to erythrocytes [372], whereas the membrane-bound isoform is anchored to MOM, ER, and plasma membrane lipid rafts [368,373,374]. Importantly, the OMM-bound CYB5R3 enzyme, ubiquitously expressed in all mammalian cells, is functionally attached to the voltage-dependent anion channel 1 (VDAC1), one of the most prevalent proteins located in the OMM [375,376].

Originally known as mitochondrial porin after its identification in yeast (1985) [377] and humans (1989) [378], VDAC was subsequently observed as a resident protein of lipid rafts in the plasma membranes of animal hearts, brains, and lungs [379] from different human cell lines, including epithelial cells, astrocytes, and neurons [380,381]. Aberrant lipid composition in neuronal lipid rafts disturbs physiological VDAC protein interactions that can affect the opening and closing of VDAC channels, resulting in oxidative stress and neuronal impairments prominent in most AD pathologies [380]. The force-from-lipid principle dictates that the opening and closing of membrane embedded channels can be propelled by the mechanical properties of surrounding lipids [382,383,384,385] and their composition. Changes to raft thickness, curvature and elasticity [291] as a result of lipid peroxidation can therefore affect physiological functions of the VDAC and CYB5R3 redox complex.

CYB5R3 enzymes form large redox centers in lipid rafts that enhance mitochondrial respiration rate and ATP production, albeit resulting in increased production of ROS [368,373,374]. Over stimulation and clustering of CYB5R3 induced oxidative stress-mediated apoptosis of cerebellar granule neurons [386]. Independent of respiratory chain activities, the ascorbate-dependent NADH: cytochrome c oxidoreductase oxidation of NADH at CYB5R3 centers in lipid rafts is also a major source of extracellular superoxide [376,387,388,389,390] that can initiate lipid peroxidation. In Wistar rats, the deregulation of CYB5R3 promptly triggers apoptosis due to the overproduction of superoxide anions at neuronal plasma membranes [368,387]. Excess NADH due to CYB5R3 redox dysfunction can close VDAC, suppressing OXPHOS and increasing glycolysis [376,391], whereas the opening of VDAC also elevates ROS from increased OXPHOS activities [41]. As the most abundant protein in the MOM, VDAC is regarded as a dynamic regulator of mitochondrial functions, interacting with over 100 proteins in health and disease [392]. VDAC opening is believed to globally control mitochondrial metabolism and ROS formation, modulating mitochondria and cellular bioenergetics [41,393]. Nevertheless, the question of whether apoptosis is associated with the opening [394] or closure [395,396] of VDAC has been highly debated [397], further emphasizing the important role of this protein in the regulation of cell life and death [392,398].

VDAC is the gatekeeper which controls the export of ATP out of mitochondria into cytosol and the import of essential respiratory substrates such as ADP and Pi into mitochondria [395,399]; therefore, VDAC opening may be instrumental in determining the fate of MLO formation, regulation, and dissolution. ATP is not only a biological hydrotrope capable of inhibiting protein LLPS and aggregation at high mM concentrations, but it has recently been observed to act as a universal and specific regulator of IDRs capable of altering physicochemical properties, conformation dynamics, assembly, and the aggregation of MLOs [45]. Not only is the preservation of lipid raft structure and composition essential for maintaining specific ion channel properties [380], the amount of cytosolic ATP is dependent upon mitochondrial synthesis and the integrity of CL enriched raft-like lipid domains in mitochondria [367,400,401,402].

The mitochondrial electron transport chain is a major ROS-generating site where complex III and mitochondrial glycerol 3-phosphate dehydrogenase can produce large amounts of redox signaling molecules such as superoxide and hydrogen peroxide to the external side of the IMM as well as the matrix [403,404]. Bis-allylic methylenes and abundant double-bonds in CL lipid chains are vulnerable targets of ROS attacks [239,405,406,407]; therefore, the lipid monolayer leaflets facing the crista lumen enriched in CL in mitochondria [228] may be subject to intense peroxidation events. Peroxidized CL could not support mitochondrial OXPHOS enzyme activities [239,408], leading to the depletion of ATP [409] that can potentiate and exacerbate the aggregation of pathological MLOs.

Melatonin is an ancient, potent antioxidant that protects lipid nanodomains from peroxidation caused by excess oxidative stress. The addition of micromolar concentrations of melatonin to rat heart mitochondria dramatically inhibited CL oxidation by tert-Butylhydroperoxide (t-BuOOH), a peroxidation promoting peroxide, reversing cytochrome c release, matrix swelling, and proton motive force (ΔΨ) collapse in treated cells [301]. The melatonin molecule is uncharged in the entire pH range [410] and contains both hydrophilic and lipophilic moieties that support its easy accumulation in all internal membranes of cells as well as other hydrophobic sites [411,412]. The exogenous supplementation of melatonin in rodents results in dose-dependent increases in all subcellular compartments, with lipid membranes exhibiting 10-fold increases compared to mitochondria [413]. The presence of both hydrophilic and lipophilic moieties in melatonin not only facilitates the efficacious scavenging of both aqueous and lipophilic free radicals [411], but also places the molecule in a unique position during evolution to protect membrane lipids from oxidative damage and potentially regulate MLOs that form at membrane surfaces in an ATP-dependent manner (Figure 1).

## 4. Melatonin Is a Potent Ancient Antioxidant That Protects ATP Levels to Regulate the Formation and Dissolution of MLOs

Melatonin (*N*-acetyl-5-methoxytryptamine) is a mitochondria-targeted molecule found in cells of all tested eukarya and bacteria [414]. Effective distribution via horizontal gene transfers may explain the discovery of ancient homologs of arylalkylamine *N*-acetyltransferase (AANAT), the enzyme responsible for the rhythmic production and release of melatonin in bacteria, fungi, unicellular green algae, and chordates [415,416,417]. In present-day vertebrates, it is estimated that ~99% of melatonin is likely not produced in the pineal gland, nor released into circulation upon pineal production [418], but is mainly synthesized and localized in mitochondria [419,420]. Photosynthetic cyanobacteria responsible for filling the earth with oxygen that led to the extinction of obligate anaerobes produce melatonin [421,422]. The presence of melatonin in primitive unicellular organisms including *Rhodospirillum rubrum* and cyanobacteria, precursors to mitochondria and chloroplasts, respectively [415,423,424,425], may have conferred protection against endogenous and exogenous oxidative stress that could readily damage biomolecules and disrupt ATP production at plasma membranes [421,425,426,427]. This unique feature implies that melatonin may have an intrinsic modulatory effect over phase separation in early organisms.

As in all eukaryotic cells of plants and animals, LLPS is also believed to be the organizing principle behind the subcellular compartmentalization of membraneless organelles (MLOs) in prokaryotic bacteria [277,428], where condensate formation is tightly correlated with ATP levels. Impaired ATP hydrolysis from reduced ATPase activity in bacteria causes droplet formation by phase separation [429,430]. Cyanobacteria, the only known prokaryote capable of water oxidation [431], has recently been shown to exhibit circadian rhythm in the formation and dissolution of MLOs that remained soluble during daylight, but became reversible, insoluble condensates at night. The formation of aggregates allows cyanobacteria to conserve energy when metabolic activities and ATP levels are lowered at night [432,433,434,435]. It is therefore not unexpected that when ATP production was disrupted, insoluble aggregates could be induced to form in cyanobacteria even during daylight by suppressing F_1_F_0_-ATP synthase or uncoupling OXPHOS with mitochondrial proton gradient inhibitors [432].

The gene sequences of cyanobacteria ATP synthase subunits are extremely similar to those in chloroplasts [436]. Embedded in the thylakoid membrane, both ATP synthase in cyanobacteria and chloroplasts (CF_0_CF_1_) control transmembrane electrochemical proton gradients for the production of ATP [437,438,439]. Similar to CL, which is synthesized from phosphatidylglycerol (PG) in all organisms [440], PG is the primary phospholipid associated with photosystem complexes that carry out electron transport reactions during oxygenic photosynthesis [441]. Both CL and PG are essential for maintaining the proper lipid composition that supports electron transport and ATP production in eukarya and prokarya, although these lipids are easily subjected to damage via lipid peroxidation [213,234,442,443,444,445,446]. The antioxidant effects of melatonin and its metabolites become particularly meaningful when the prevention of CL peroxidation by hydroperoxyl in mitochondrial membranes can affect the formation and dissolution of biomolecular condensates (Figure 1).

### 4.1. Melatonin Metabolite 3-OHM Inhibits Lipid Peroxidation by Hydroperoxyl Radical

Melatonin and its secondary, tertiary, and quaternary metabolites actively scavenge potent free radicals [317,426,447] including hydroxyl radicals [448], singlet oxygen [449,450], hydrogen peroxide [451], nitric oxide [452,453,454], and peroxynitrite anions [455] via different antioxidant mechanisms such as direct radical trapping in Type I antioxidant reactions and inactivating hydroxyl radicals (^•^OH) through the sequestration of metal ions and deactivating ^•^OH during Fenton-like reactions in Type II antioxidant reactions [456]. In addition, melatonin and its metabolites collectively preserve the chemical integrity of biomolecules from oxidative stress via Type III antioxidant cellular repair processes and Type IV antioxidant reactions that can enhance antioxidant enzymes and inhibit pro-oxidant enzymes [456].

A recent study that analyzed the mechanistic interactions between melatonin and ^•^OH employing density functional theory found that one molecule of melatonin effectively scavenged two ^•^OH radicals to produce the stable footprint metabolite, cyclic 3-hydroxymelatonin (3-OHM) [457], in perfect agreement with mechanisms reported in prior experimental and theoretical studies [448,458,459,460]. 3-OHM has been shown to react with hydroperoxyl radicals (^•^OOH) at rates 98.4 times faster than Trolox in aqueous solution [459]. Trolox is a water-soluble, cell-permeable analog of vitamin E with high radical scavenging potential often used as a yardstick for measuring antioxidant capacities in vitro. Trolox resides mainly in the aqueous phase; therefore, it has been observed that Trolox and other water-soluble antioxidants exhibit reduced scavenging activity if radicals are produced within hydrophobic cores of lipid membranes [461]. Melatonin accumulates in all of the internal membranes of cells as well as other hydrophobic sites [412]; therefore, this antioxidant may be uniquely positioned for quenching lipid peroxidation by ^•^OOH and other free radicals that penetrate deep into lipid molecules.

### 4.2. Melatonin Is Preferentially Located at Hydrophilic/Hydrophobic Membrane Interfaces

All biological cell membranes comprise amphipathic lipid molecules with hydrophilic heads and hydrophobic tails that naturally form bilayers with headgroups oriented towards an aqueous environment and tails facing each other [462]. The melatonin molecule is uncharged in the entire pH range [410] and, accordingly, in laboratory environment, the “hydrophobic” molecule is dissolved poorly in water [463] except when solubilized in pure aqueous medium by specific methodology that polarized the pyrrole ring to facilitate hydrogen bonding of the N–H group [464]. The unique ability to form strong H-bonds with hydrophilic lipid headgroups allowed nonpolar melatonin to be preferentially located at hydrophilic/hydrophobic interfaces, with complete solubility observed at the interfaces between polar and lipophilic nanodomains in reversed micelles [320]. The presence of both hydrophilic and lipophilic moieties in melatonin facilitates the scavenging of both aqueous and lipophilic free radicals [411], especially ^•^OH [448] and ^•^OOH, the two most prevalent ROS responsible for the chain oxidation of unsaturated phospholipids [465,466] in the membranes of cells and mitochondria [467,468].

### 4.3. Melatonin Metabolite Free Radical Scavenging Cascades Rescue Cardiolipin from Hydroperoxyl Radicals (^•^OOH)

Lipid peroxidation, a physiological process in all aerobic cells [469], is a cascading chain reaction that begins with the abstraction of allylic hydrogen from adjacent lipid molecules by free radicals such as ^•^OOH and ^•^OH and terminates with reactive aldehyde end products such as malondialdehyde (MDA) and 4-hydroxynonenal (HNE) [212,470,471,472,473]. Both ^•^OOH and ^•^OH are derived from ubiquitous superoxide radicals (O_2_^•−^) generated from the one-electron reduction of oxygen (O_2_) that may be catalyzed by nicotinamide adenine dinucleotide phosphate oxidase (NADPH oxidase) during respiratory bursts [474] and/or electron leakage during mitochondrial electron transport [403]. Due to its low rate constant values below ~102 L·mol^−1^·s^−1^ [475], O_2_^•−^ behaves more similarly to an unimpressive reductant (E°′(O_2_/O_2_^•−^) = −0.33 V) than an oxidant (E°′(O_2_^•−^/H_2_O_2_) = 0.93 V) [472,476,477,478] which reacts at a much slower pace with the tested phospholipids compared to ^•^OOH [466,479]. Hydroperoxyl (^•^OOH or HO_2_^•^), also known as a perhydroxyl radical, is a chemically active, protonated form of superoxide radicals (O_2_^•−^) [480], engaged predominantly as intermediates for the disproportionation of O_2_^•−^ into hydrogen peroxide (H_2_O_2_) which then can further be transformed via Fenton’s/Haber–Weiss reactions [481] into ^•^OH, possibly the most reactive and mobile species of oxygen that interacts with almost all molecules in cells [212,481]. Even though at neutral pH ^•^OOH exists primarily as the less reactive O_2_^•−^, where the ratio of protonated ^•^OOH to anionic O_2_^•−^ is ~130:1 (less than 1%), ^•^OOH can be a potent initiator of lipid peroxidation [465,466].

When reacting with phospholipids, the advantageous free energy profile of −8.5 kJ/mol free energy minimum relative to the aqueous phase allowed ^•^OOH to accumulate at lipid headgroup membrane–water interface at concentration enhancement of over one order of magnitude [295]. Multi-level atomistic simulations for interactions of ^•^OH, ^•^OOH, and H_2_O_2_ with polar headgroups of phospholipid bilayer revealed that all three species traveled deep into the water layer to reach phospholipid biomolecules, oxidizing hydrophilic headgroups before hydrophobic tails [482], with ^•^OOH staying adsorbed for the longest duration at headgroup regions [295]. The headgroup of CL is fully ionized as a dianion in the physiological pH range [483], supporting its unique, optimal functionality as a “proton trap” that promotes mitochondrial respiratory enzyme activities [484]. 

The strong negative curvature of cristae in the IMM is primarily sustained by the distinct molecular geometry of CL with its smaller, elongated, conical-shaped, double-phosphate dianonic headgroups that increase lateral pressure within the acyl chain regions and stabilize cylindrically curved, tubular cristae structures [223,485,486]. In large unilamellar vesicles (LUVs) comprising similar lipid properties as the IMM, the addition of a typical concentration of 25% negatively charged, dianonic CL lowered pH at the membrane interface to ~3.9, compared to the bulk pH of 6.8 normally found in mitochondrial intermembrane space [487] and 7.7 in the matrix space [488]; in contrast, LUVs with mono-anionic lipids only reduced the pH to ~5.3 at the membrane interface [487]. The reduced pH at the membrane interface from CL, linearly associated with increased proton (H^+^) concentration (~700 to ~800) [487], is the reason why ATP production is doubled in mitochondrial models with cristae compared to those without [409]. At the same time, the increased H^+^ concentration at membrane surfaces may cause accumulation of ^•^OOH, the protonated form of O_2_^•−^ [480].

^•^OOH remains adsorbed at polar headgroups longer than other ROS tested [295]; therefore, a low pH at membrane interface that is favorable for enhanced ATP synthesis could also initiate peroxidation cascades. As such, even though the proper functioning of CL is prerequisite for optimal mitochondrial respiration and ATP production, peroxidation of CL in mitochondria is an inevitable, natural, physiological process that can deteriorate pathologically [239,241,405,489,490,491,492,493,494,495,496,497,498] unless properly counterbalanced by the continuous synthesis [420] and/or uptake of high levels of melatonin. Melatonin is known for its role in maintaining systemic energy homeostasis [499]. In the mitochondria of brown and beige adipose tissue, CL biosynthesis is robustly induced upon cold exposure [500,501] because CL can bind tightly to uncoupling protein 1 (UCP1), stabilizing its conformation and enhancing functionality [502]. The ability of melatonin to protect CL from peroxidation may account for the increased thermogenic response in Zücker diabetic fatty (ZDF) rats via the restoration of UCP1 mRNA expression, increased mitochondrial mass and brown adipose tissue (BAT) weight, as well as enhanced mitochondrial OXPHOS activities in complex I and IV [503].

### 4.4. Melatonin May Regulate Glycolytic G Bodies by Increasing ATP

As early as 2002, melatonin was found to increase mitochondria OXPHOS activity and elevate the production of ATP [504]. Recent experimental and theoretical studies have presented different mechanisms explaining how melatonin may function as a glycolytic, such as stimulating the SIRT3/PDH axis in vitro to reverse the Warburg phenotype in lung cancer cells [505], converting cells to a healthy phenotype by inhibiting hypoxia-inducible factor-1α to encourage OXPHOS over glycolysis induced by hypoxic conditions [506], downregulating pyruvate dehydrogenase kinase (PDK) to increase acetyl CoA synthesis [507,508], or elevating α-ketoglutarate (α-KG) levels in macrophages to promote M2 polarization that favors OXPHOS over glycolysis [509,510].

Interestingly, in *Saccharomyces cerevisiae* and human hepatocarcinoma cells challenged with hypoxic stress, the non-canonical RNA-binding proteins in glycolytic enzymes have been observed to promote phase separation [511] that facilitate and maintain the assembly of glycolysis enzymes into cytoplasmic, membraneless glycolytic G bodies that increased glycolytic output during hypoxia [512]. Melatonin is able to increase ATP concentration in cells [503,504,505]; therefore, the switch between OXPHOS and glycolysis could possibly be part of the effect where high ATP concentration dissolves MLO aggregations. Molecular dynamics simulation experiments revealed that the propensity for self-aggregation enhanced the role of ATP as a hydrotrope, preferentially binding to polymers to unfold hydrophobic macromolecules and disrupting the aggregation process of hydrophobic assemblies via the introduction of charges to the macromolecules [513]. These results may explain previous observations where a high cytosolic ATP:ADP ratio readily suppressed glycolysis, whereas the closure of VDAC channels resulting in lower ATP:ADP ratios in cytosol activated glycolysis in vitro [514]. Alterations to the glycolytic pathways are often observed during the early stages of neurodegenerative diseases where mitochondrial dysfunction and reduced ATP levels may contribute to protein aggregation [515]. Increasingly, the pathogenic aggregation of MLOs such as stress granules, p53, FUS, TDP-43, and tau exhibiting dysregulated LLPS is believed to play a major part in the development of neurodegeneration and cancer [12,516,517,518].

## 5. Melatonin May Attenuate the Stress-Induced Aggregation of Pathological MLOs via Post-Translational Modification and RNA Modification in an ATP-Dependent Manner

Biomolecular condensates containing protein, RNA, and other nucleic acids [1] are formed by LLPS under changing endogenous or exogenous conditions, including stress responses [3] and signal transduction [4,5], as well as genome expression, organization and repair [6]. In eukaryotes, gene transcription is executed by transcription factors, including p53 [519,520], TDP-43 [521,522], and FUS [523], containing IDRs that form condensates to compartmentalize and assemble necessary factors [6,524]. Transcription is essentially a nonequilibrium process that employs RNA products to provide a two-way dynamic feedback control in the regulation of electrostatic interactions in transcriptional condensates [108,525,526] where RNA products recruit proteins to form molecular scaffolds driving phase separation, whereas many essential RNA processes such as transcription, transport, and metabolism are regulated by phase separation [527]. Under stress, different RNA species are often incorporated by different MLOs because unique RNA–protein interactions can define biophysical properties of MLOs such as stress granules [528,529]. Cells rely upon RNA to regulate condensates because RNA molecules contain powerful electrostatic forces due to the high negative charge densities buried in their phosphate backbones [530,531,532]. Therefore, a low level of RNA with a negative charge could interact with positively charged proteins to promote phase separation and the formation of transcriptional condensates, whereas high levels of negatively charged RNA could repel proteins with a positive charge to dissolve condensates [525].

Cells also employ post-translational modifications (PTMs) to induce non-equilibrium thermodynamic chemical reactions in order to tune the molecular interactions of key condensate components where external energy input drives reactions out of equilibrium to control the size and number of MLOs [533]. PTMs, including phosphorylation, acetylation, glycosylation, methylation, ubiquitination, and SUMOylation [11,79], may function as phase-separation on–off switches [60,534] or rheostats that actively adjust the dynamics of LLPS during condensate formation [79,535]. Under different cellular conditions, including stress, PTMs can either promote or suppress LLPS by modulating protein valency and interaction intensities [79,351,536], as well as recruit or exclude proteins from condensates [537,538].

### 5.1. Cellular Stress and Mutations Drive Dysregulated LLPS to Form Pathological Aggregates in Neurodegenerative Disorders

Cellular stress in eukaryotes activates defense mechanisms such as stress granules (SGs) that can promote either survival or apoptosis [539]. Integral to cellular stress management adaptations [540], SGs are membraneless, cytoplasmic complexes comprising non-translating mRNA and RNA-binding proteins (RBPs) [541] assembled from RNA–RNA interactions [542]. Type I stress, including hypoxia, heat-shock, and arsenite [539], can induce the formation of SGs to increase cell survival by reprogramming cellular metabolism through the modulation of cytoplasmic mRNA functions [540,541]. Oxidative stress induced by tellurite has recently been documented to assemble bona fide cytoplasmic and nuclear SGs in vitro [543]. Under oxidative stress, increased SGs in senescent cells is one of the key post-transcriptional gene expression regulators [544]. The rapid and dynamic range of gene expressions in immune cells may also be regulated by mRNA translation control modulated by SGs [545]. Interestingly, SGs have been found to host many of the proteins that contain long segments which are intrinsically disordered [546,547] and capable of LLPS to form pathological aggregates [548,549] associated with diseases such as neurodegeneration [550,551] and cancer [552]. It has been proposed that the aggregation of pathological TDP-43, FUS, and tau is processed through the stress granule pathway [553]. The fact that degenerative diseases have been associated with IDR-containing pathological aggregates of p53, tau, TDP-43, and FUS [554,555,556,557], which are also important transcription factors [519,520,521,523] associated with SGs, emphasizes the relevance in the interactions between these MLOs for the dynamic assembly of SGs under stress conditions inhibiting the initiation of mRNA translations, and the necessity of their timely, rapid disassembly upon stress removal [558].

Under cellular stress conditions, phosphorylation can initiate the formation of SGs [559] and also increase tau-phosphorylation which, in turn, appears to increase SG formation [560,561]. Once formed, the subsequent colocalization and interactions between phosphorylated tau and RNA-binding proteins abundantly present in SGs could further enhance the aggregation of insoluble cytotoxic neurofibrillary tangles (NFTs) [553,562,563]. Under cellular stress, TDP-43 and FUS are released from the nucleus where they reside under physiological conditions into the cytoplasm and assemble with SGs [562]. The aggregation of RBPs such as FUS, TDP-43, and even p53 [564] in the cytoplasm has been reported to be linked to phase separation which is regulated by RNA concentration. Both FUS and TDP-43 contain intrinsically disordered, prion-like, low-complexity domains that are soluble in the nucleus due to high levels of RNA, but phase-separate into aggregates driven by lower RNA concentrations in cytoplasm [28,57,565,566]. In the same manner, a low RNA:protein ratio (1:50) caused the formation of large amorphous p53 aggregates in vitro, whereas a higher ratio of 1:8 inhibited aggregation [567,568]. If stress is not resolved in a timely manner, aggregations may become irreversible and insoluble [569]. Prolonged physiological stress and/or mutations in genes coding for TDP-43 [570,571] and FUS [572,573] can lead to enhanced stress granule formation, which could accelerate the pathological aggregation of these proteins in neurodegenerative diseases [553,574,575]. A single substitution of only one residue in a protein sequence, commonly referred to as missense mutation, can also affect macromolecular stability, cellular localization, and perturb macromolecular interactions [576] during LLPS.

Missense mutations associated with diseases are found mostly within IDRs [577]. These mutations in IDRs can cause the dysregulation of LLPS by changing the threshold concentration for condensate formation [578,579,580], modulating the exposure of the aggregation-prone regions [577], and interfering with RNA interactions [27]. IDR mutations are capable of disrupting phase separation in important cellular processes, turning dynamic liquid droplets into aberrant fibril aggregates [581,582,583] to cause mislocalization or the gain/loss of functions [27,584]. TDP-43, an important RNA-binding protein, is the major disease protein where the pathological form is hyperphosphorylated and ubiquitinated in ALS [585]. The C-terminal domain of TDP-43 is a prion-like domain (PLD) [586] which is intrinsically disordered [587] and harbors almost all ALS-causing mutations that drive the LLPS of TDP-43 to associate with stress granules to form pathological aggregates or amyloid fibrils [588,589]. These mutations disrupt LLPS by inhibiting interaction and helical stabilization to enhance aggregation and disrupt protein interactions [590,591,592,593].

Intriguingly, ATP has recently been reported to exhibit a unique biphasic relationship with TDP-43 PLD. At a molar ratio of only 1:25 (PLD:ATP), TDP-43 PLD was induced to undergo LLPS to start forming liquid droplets in a dose-dependent manner where many droplets could be produced at a 1:100 molar ratio. Further increases in ATP, in contrast, led to a reduction in droplet formation. At 1:750, only a few droplets could be detected, and at 1:1000, all droplets were disassembled by ATP. Importantly, in the absence of ATP, TDP-43 PLD was unable to phase-separate into droplets [53]. Neurons have been reported to produce up to 5 mM of ATP in cytoplasm through glycolysis [594], whereas the cytoplasmic concentration of TDP-43 in neurons may be several thousand times lower [53,569,595,596]. Therefore, under physiological conditions, ATP could regulate most IDRs by modulating physicochemical properties, conformations, dynamics, LLPS and aggregation [53]. At physiologically relevant concentrations, ATP has been reported to bind tightly with TDP-43, enhancing thermodynamic stability and prohibiting LLPS-induced pathological fibrillation [597].

Mutations in fused in sarcoma (FUS) are associated with ALS pathology, and are believed to be a major cause behind familial ALS [598,599]. Under physiological conditions, FUS is a multifunctional, DNA-/RNA-binding protein responsible for maintaining genomic stability, RNA metabolism, and stress responses [600]. Under stress conditions, wild-type (WT) FUS may remain nuclear whereas mutant mislocalized FUS in cytoplasm are assembled into stress granules [47,600]. WT FUS exhibit dynamic RNA interactions whereas mutants display altered, static interactions with RNA, leading to a buildup of aggregates in aberrant phase separations [27]. In addition, mutant FUS exhibit a gain-of-toxic mechanism that delay the assembly and alter the structure and dynamics of SGs [572].

Physiological FUS is a transcription factor [523] which has been identified to regulate circadian gene expression via a novel feedback effect [601]. FUS mutations interfere with RNA metabolic pathways and suppress protein translation [602]. Mutant FUS (R52aC) disrupted the feedback effect to lower the expression of the E box-containing core circadian gene Per2 by binding to RNA-/DNA-binding splicing factor protein (PSF) [601]. Similarly to TDP-43, ATP has also been identified to enhance the LLPS of FUS at low concentrations, but dissolves FUS aggregates at higher concentrations [51]. Phosphorylation is an important post-translational modification used by cells to regulate transcription factors [603,604,605] including FUS. In yeast models, phosphorylation of the low-complexity domain in FUS not only disrupted phase separation, but reduced toxicity and the prion-like aggregation propensity of FUS [580]. The synthesis of melatonin in neuronal mitochondrial [420] fulfils a range of important functions, including balancing oxidative stress to maintain relevant physiological levels of ATP and possibly to ensure the proper execution of PTMs such as the increase in phosphorylation to enhance neurogenesis in the mouse subventricular zone (SVZ) that has been reported in experimental studies [606,607].

### 5.2. Melatonin Inhibits/Disaggregates Pathological Tau Neurofibrillary Tangles and May Regulate the Phosphorylation of Tau in Neurodegenerative Disorders

Phosphorylation is one of the most important PTMs that can control the assembly/disassembly of MLOs [608] as well as stabilize or destabilize MLOs including G bodies [512] and p53 [609]. Cells rely on phosphorylation as rapid, reversible responses to different stimuli by changing the physicochemical properties of proteins during phase separation multivalent interactions [79,538]. Phosphorylation establishes covalent bonds between phosphoryl and amino acid hydroxyl groups using the terminal phosphate group in ATP [610]. The phosphoryl group is negatively charged; therefore, the attachment turns the polar, uncharged residue into a negatively charged amino acid [60]. In theory, charged residues can prevent protein aggregation and increase the solubility of water-soluble proteins [611]. Indeed, phosphorylation has been observed to modulate the size of MLOs [361,535], disassembling synapsin 1 droplets [612] and preventing membrane-attached zona occludens (ZO1) from phase-separating into droplets that form tight junctions in tissues [613]. Similarly, in *C. elegans*, phosphorylation also promoted IDR granule disassembly, whereas dephosphorylation promoted granule assembly [614]. Under different cellular conditions including stress, PTMs can either promote or suppress LLPS by modulating protein valency and interaction strengths [79,351,536], as well as recruit or exclude proteins from condensates [537,538].

The ATP-dependent DEAD-box helicase [307] DDX3X responsible for initiating NLRP3 inflammasome aggregation is dependent upon phosphorylation-associated IFN promoter stimulation [304,310,615,616]. When the conserved, eukaryotic, integrated stress response (ISR) pathway is activated by external stress stimuli including hypoxia, nutrient deprivation, viral infections, as well as intrinsic ER stress [617], the phosphorylation of eukaryotic translation initiation factor 2 alpha (eIF2a) on Ser51 [618,619] triggers the formation of stress granules as adaptive homeostatic responses to promote survival and restore homeostasis [620,621,622,623] via mRNA translational modification that may involve the repression of protein synthesis [541,624,625,626]; however, dephosphorylation of eIF2a blocks the ISR pathway [627]. The formation of SGs via eIF2a-dependent and -independent pathways [628] during stressful conditions allows cells to conserve energy by reducing global protein synthesis that may prevent the accumulation of harmful misfolded proteins, while preserving the selective translation of genes that assist in survival and recovery [629]. Results from in vitro experiments suggest that SGs form phase-separated, dynamic structures from IDR-containing proteins that can mature over time into stable structures [26,581,582,622,630]. The clearance of stress granules may be carried out by autophagy [631] or the disassembly of shells and cores via an ATP-dependent process [622]. In AD pathology, the hyperphosphorylation of tau proteins thermodynamically facilitates the oligomerization of pathological intracellular neurofibrillary tangles [632,633].

AD is associated with the aggregation of Aβ as well as the intracellular deposition of neurofibrillary tangles (NFTs) of tau, a major neuronal protein with important physiological functions of stabilizing and promoting the assembly of microtubules (MTs) in the central nervous system (CNS) [634,635]. The intrinsically disordered, highly soluble nature of tau in solution facilitates binding to MTs [636]. Under physiological conditions, tau readily converts between soluble monomers and phase-separated droplets that disassemble quickly. Physiological tau droplets support important biological functions specific to the cellular compartments where they are formed [637,638], such as myelination [639,640], axonal transport [639,641], motor function [642], learning and memory [643], neuronal excitability [644], as well as glucose metabolism [645,646,647], DNA protection [648], and gene transcription [520]. In neurons, phase-separated tau droplets enhance the nucleation of MTs, promoting tubulin polymerization by decreasing critical concentration [649,650]. Physiological tau condensates not only stabilize dynamically unstable MTs [651], but can form islands on the surfaces of MTs, protecting them from severing enzymes [652,653]. However, phosphorylation-dependent LLPS that forms physiological tau droplets can also initiate the production of tau amyloids upon the coacervation of positively charged microtubule-binding domains with negatively charged molecules [29,636,637,654]. The deposition of fibrillar hyperphosphorylated misfolded tau aggregates in the brain is accepted as a key biomarker for AD and tauopathies [655,656].

LLPS of tau has been demonstrated to promote amyloid aggregation [657]. Tau can undergo electrostatically propelled LLPS with itself in simple coacervation or with a large number of RNA polyanions in complex coacervation [657,658,659,660]. Experimental model systems have revealed that the pathological aggregation of tau is predominantly mediated by hydrophobically driven LLPS which leads to the dehydration of interfacial water, further amplifying hydrophobic associations [661]. Such strong hydrophobic attractions are believed to be the cause for hyperphosphorylated tau in tauopathies [662,663,664,665]. Even though the hyperphosphorylation of tau is a transient, reversible physiological process, in neurodegenerative disorders such as AD, abnormal hyperphosphorylation of tau is resistant to dephosphorylation and proteolysis [666,667], often resulting in a 3–4-fold increase in accumulation compared to normal brains [668,669,670]. Abnormally hyperphosphorylated tau is disassociated from MTs and loses its MT-stabilizing physiological functions [662,671]. The pathogenic phosphorylation of tau may also be site-specific; the phosphorylation of multiple tyrosine residues including Tyr-310 has been demonstrated to inhibit tau aggregation [672]. Even though hyperphosphorylated tau precedes the appearance of NFTs [673], altering its important physiological role in DNA protection [648,665,674,675], there are unanswered questions surrounding the phosphorylation and hyperphosphorylation of tau [676].

Tau phosphorylation has been proposed as a neuroprotective mechanism [677] where phosphorylated tau sequesters redox active heavy metals [678,679] and NFTs may provide antioxidant defense against oxidative damage [680,681], whereas hyperphosphorylated tau protects neurons from apoptosis [682,683]. An experimental study that phosphorylated specific microtubule binding sites of tau, including K18 and pS356/pS262 and employing a total chemical synthetic approach, discovered that the hyperphosphorylation of K18 inhibited aggregation, seeding activity, binding to microtubules, and microtubule polymerization [684]. These results support the hypothesis that the phosphorylation of tau may be a protective mechanism, and contradict the prevailing concept of the pathogenic nature of hyperphosphorylated tau [685,686,687] which could impair cell viability [688] and accelerate the progression of cognitive impairments [655,689,690,691].

The use of melatonin in neurodegenerative disorders has been extensively studied and reviewed [333,692,693]. Numerous experimental and theoretical studies successfully demonstrated the high efficacy of melatonin in attenuating various pathological effects of tau hyperphosphorylation, employing different mechanisms, including: activating the phosphorylation of p-Akt-Ser473 in a PI3K-dependent manner [694,695]; inhibiting GSK3β-activated tau hyperphosphorylation [696,697,698] to decrease Aβ_1–42_-induced memory impairment, synaptic disorder, and tau hyperphosphorylation-associated neurotoxicity in C57BL/6N mice [699]; restoring autophagic flux, inhibiting caspase-3 activation, and reducing abnormal protein aggregation to ameliorate tau-pathology-related symptoms such as oxidative stress, neuroinflammation, cognitive impairment, cell death, and tau hyperphosphorylation in experiments using humans/rats ex vivo (10 μmol/L melatonin) and mice in vivo (10 mg/kg melatonin) models [700]; and decreasing calpain expression/activation, GSK-3β activation [697]. Melatonin decreased ER stress induced by kainic acid, easing tau hyperphosphorylation and memory impairment in mouse models, although substitution with vitamin E did not produce the anticipated antioxidant effects on the reduction in ER stress [701]. Even the use of the melatonin receptor agonist agomelatonin was able to prevent tau protein phosphorylation and oxidative damages induced by Aβ_25–35_ in pheochromocytoma (PC12) cells by activating melatonin-PTEN/Akt/GSK3β signaling [702]. The majority of these experiments showed an association between the reduction in tau hyperphosphorylation-related neurotoxicity and activation of the Akt-PI3K/GSK3β signaling pathway by melatonin. The fact that PI3K is a pro-survival, pro-stress-granule kinase that promotes the assembly of stress granules [703] adds an additional layer of complexity to the mechanisms employed by melatonin in the attenuation of tauopathies.

Even though melatonin is able to ameliorate tau-pathology-related symptoms such as oxidative stress, neuroinflammation, cognitive impairment, cell death, and tau hyperphosphorylation in vivo [700], the fact that Luengo and colleagues supplemented C57BL/6J male/female mice with melatonin only after all symptoms of tauopathy were firmly established (7–28 days) may imply that a further promotion of SG formation via activation of AKT-PI3K was possible, potentially increasing the additional pathological aggregation of tau because the colocalization and interactions between phosphorylated tau and RNA-binding proteins abundantly present in SGs may enhance the assembly of insoluble cytotoxic NFTs [553,562,563]. Similar to the multiple pathways and mechanisms employed by melatonin in effecting the switch from glycolysis to OXPHOS [506,507,508,509,510], there may yet be another compelling reason that could explain how melatonin at pharmacological doses (10 mg/kg in vivo) [700] exerts neuroprotective effects in tauopathy.

An in vitro study on Neuro2A cells reported that melatonin at 10 μM concentration reduced intracellular ROS levels induced by tau aggregate treatment, and at 50 μM, melatonin reduced phospho-tau as well as GSK3β mRNA and subsequent protein levels. Melatonin increased cell viability in tau-exposed neurons in a dose-dependent manner, with 80% viability observed at 20 μM melatonin and a complete reversal at 200 μM, compared to only a 60% viability in controls without melatonin [704]. In an earlier study, the same group had reported that melatonin at strengths between 200 and 5000 μM failed to deter the aggregation of full-length tau. However, distinct morphology of small, broken tau fibrils were seen in the presence of either 1000 [705] or 5000 μM [352] melatonin. Furthermore, 5000 μM melatonin disaggregated tau fibrils by 54%, whereas 100 μM achieved only a ~14% effect [352]. It is possible that melatonin interacts with histidine residues to destabilize the assembly of aggregates [352] in a manner similar to how it disrupts salt bridges in Aβ, because tau phosphorylation alters side chain conformations through the formation of a network of salt bridges [706]. Salt bridge interactions were also observed in Aβ-mutated tau complexes assembled from Aβ peptides and mutated tau [707]. Earlier studies have reported that 300 μM melatonin interacted with hydrophobic segments in Aβ_1–40_ and Aβ_1–42_ to inhibit the formation of β-sheet and/or amyloid fibrils [708], and the inhibition of β-sheet and amyloid fibrils in samples containing 250 μM of Aβ_1–40_ and Aβ_1–42_ with only 100 μM of melatonin could not be replicated in control experiments using a potent free radical scavenger *N*-t-butyl-a-phenylnitrone (PBN), or a melatonin analog 5-hydroxy-*N*-acetyl-tryptamine (NAT) [337]. Even though melatonin could dissolve fibrils [709] by disrupting inter-peptide salt bridges between side chains Asp23 and ly28 [710,711] critical to β-sheet formation [712], the concentrations of 1000 [705] or 5000 μM [352] required to disassemble tau fibrils are significantly higher than the 100–300 μM melatonin used to inhibit β-sheet and amyloid fibrils [337,708], or the complete reversal of cell viability in tau-exposed neurons achieved with only 200 μM melatonin [704]. More importantly, if PI3K-induced SG activation and tau hyperphosphorylation serve pro-survival functions, then there should yet be another mechanism that could convert physiological phase-separated tau droplets into highly ordered pathogenic fibrils implicated in various neurodegenerative disorders that may be rescued by the presence of melatonin.

### 5.3. Melatonin May Ameliorate Pathological Tau Fibrillation by Protecting Lipid Composition in Membranes and Lipid Rafts

MLOs are found abundantly in the nucleus, cytoplasm, and on the membranes of almost all eukaryotic cells [82], where they perform important biological functions that may regulate receptor/transmembrane protein signaling via the alteration of protein binding affinity and the modulation of local environments [86]. As such, membranes become indispensable to LLPS due to their ability to concentrate and change protein thresholds during phase separation [79], facilitated largely by lipid raft signaling [96]. Alterations in membrane fluidity and lipid composition that cause dysfunctional signaling in lipid rafts have been associated with neurodegenerative disorders [89,90,97,713]. Neuronal membrane lipid rafts, co-localized with several microtubule proteins, have been observed to maintain stability and integrity in mature cortical neurons, where the disruption of raft signaling by exogenous agents (MBC, D-PDMP) causes rapid neuritic retraction that precedes neuronal death [714]. The association of tau with plasma membranes appears to be regulated by phosphorylation [715,716], where underphosphorylated tau-proline-rich regions induce membrane localization [717], and increase the phosphorylation-initiated disassociation of tau from membranes, potentially resulting in tau hyperphosphorylation and the eventual assembly of insoluble pathogenic fibrils [718].

Mutations in the Niemann–Pick type C (NPC) gene cause disturbances in cholesterol metabolism in lipid rafts [719], where a dramatic reduction in membrane raft cholesterol in NPC1-deficient cells leads to the hyperphosphorylation of tau at multiple sites [720]. Experimental evidence from a mutant human tau and APOe knockout (htau-apoe^−/−^) mouse model demonstrated the formation of tau filaments elevated intraneuronal unesterified cholesterol, which may result in a vicious circle where tau fibrils alter cholesterol homeostasis and disturb cholesterol metabolism which continues to promote tau pathology [721]. Physiological tau proteins are flexible, highly charged, and soluble, and can be extremely active on membrane surfaces, interacting favorably with anionic lipids at air–water interfaces [74]. A recent in vitro study revealed that tau–anionic lipid membrane interactions catalyzed the misfolding and assembly of tau, transitioning from random coil conformations into β-sheet aggregates that fueled tau fibrillation and deposition [74]. The binding and insertion of tau into anionic lipid membranes not only structurally compacted and misfolded tau into extended β-sheet aggregates, but disrupted lipid packing, inducing membrane morphological changes [74] including membrane roughness [722]. As a result of tau association, well-defined circular liquid-condensed (ordered) L_c_ domains [723] in anionic 1,2-dimyristoyl-sn-glycero-3-phospho-(1′-rac-glycerol) (DMPG) monolayers used as mimics for anionic lipids in neuronal cells became less defined and subsequently fused with other L_c_ domains [74]. Increased unesterified cholesterol in membranes from tau fibrillation [721] may cause L_c_ domains to become more fluid, mechanistically deforming membrane bilayer structure [724]. Melatonin has been reported to interact with cholesterol [321], binding competitively to lipid molecules to displace cholesterol [319]. L_c_ domains that have become more fluid will transition into L_o_ lipid raft domains [721,724] that could potentially potentiate the formation of enlarged lipid rafts (inflammarafts) [196,198].

Melatonin may regulate lipid dynamics and composition, modifying the lipid hydrocarbon chain order to promote phase separation in ternary membrane models [318,319], as well as preserving nanoscopic lipid raft domains by stabilizing lipid L_o_–L_d_ phase separation over a range of temperatures [350]. The ability of melatonin to penetrate and re-order lipids in membranes provides insight into its neuroprotective effects against tauopathy. Tau interacts favorably with anionic lipids at membrane interfaces [74]; therefore, the accumulation of melatonin in electron-dense anionic headgroup regions to form fluid domains that enrich lipid membranes [341] can potentially disrupt tau–lipid interactions. Indeed, in addition to dissolving tau fibrils, the addition of 1000 μM melatonin reversed all membrane roughness induced by tau aggregates in neuro2A cells in vitro [705,722,725].

The tumor suppressor p53 has been found to interact with tau and Aβ, forming pathological aggregates that result in the mislocation and impairment of its essential physiological DNA repair functions [726,727,728]. p53 has been seen to cause a shift in membrane phospholipids from mono-unsaturated acyl chains towards saturated phospholipid species that may potentially contribute to cell survival [729]. However, gain of function in p53 has also been observed in tumor cells with altered lipid raft composition comprising higher cholesterol levels [730]. The influence of melatonin over tumor-suppressor p53 may also explain its effectiveness against tauopathies.

### 5.4. Melatonin Regulates p53 and Other Biomolecular Condensates through the ATP-Dependent Ubiquitin–Protease System in Neurodegenerative Disorders

Often called the “guardian of the genome” [731], p53 is arguably the most studied mammalian transcription factor [732,733] because it maintains genomic stability by inducing the transcription of thousands of genes that may regulate the cell cycle in heterogeneous responses to different stimuli, allowing cells to adapt to varying types and levels of stress [733,734,735]. After its initial discovery in 1979 [736], p53 was intensely studied for the loss of its apoptotic, tumor-suppressing capacities [737,738,739] through inactivation by frequent mutations detected in different cancers [740,741,742]. Continued explorations of this gene have led to increased understanding of the complexity in its ability to promote survival and growth [743] through the regulation of metabolic and antioxidant pathways [744] in addition to cell elimination [745]. p53 was found to induce the expression of glutaminase 2 (GLS2) under physiological conditions to enhance mitochondrial respiration, ATP production, and antioxidant defense that protected cells from oxidative stress [746]. However, under a highly stressed environment with increased ROS that induced DNA damage, p53 suppressed the Nrf2-dependent activation of antioxidant genes, initiating cell cycle arrest and apoptosis to eliminate unrepairable damages [747]. p53 has been found to be enriched in DNA damage response effectors such as 53BP1, and the aberrant phase separation of 53BP1 impaired the activation of p53, preventing proper DNA recognition and repair [748]. Mechanisms including transcription [519], PTM [749,750,751], and degradation by the ubiquitin–protease system (UPS) [752,753,754,755,756] can fine-tune the regulation of p53 [757].

More than 50% of all human cancers have been associated with missense [758] and/or synonymous mutations of the p53 gene [742,759,760,761], where close to 95% of these mutations are located in the DNA-binding domain (DBD) [12,742,762,763,764,765] which is regulated by interactions with the extensive intrinsically disordered regions of the C- and *N*-termini that flank the DBD [547,766,767,768]. IDRs are believed to modulate LLPS [557]; therefore, it is not surprising that aggregates of both wild-type and mutant p53 have been observed in cancer cells and tissues and are regarded as a hallmark for p53 inactivation [769]. In addition, the formation of amyloid oligomers and fibrils in the DBD of p53 may produce prion-like characteristics [770,771,772] that can result in the gain or loss of functions [773]. More importantly, p53 stabilization through activation is tightly regulated by the UPS, which degrades p53 by default. Nevertheless, this “default degradation” process can be evaded through stabilization of the intrinsically disordered C- and *N*-termini of p53 [754].

#### 5.4.1. Aberrant Phase Separation/Droplet Formation May Cause Pathological Prion-like Aggregation and Inactivation of p53 in Neurodegenerative Disorders

Recent in vitro studies have demonstrated that formation of highly fluid p53 droplets at neutral and slightly acidic pH, and a low-salt environment is mediated by its *N*- and C-termini disordered domains [609]. The DNA binding domain (p53C) of p53 mutants also undergoes LLPS but evolves at a faster speed than wild-type p53C into solid-like phase transitions, resulting in the formation of amyloid-like aggregates [517]. Molecular crowding agents such as polyethylene glycol (PEG) can promote LLPS droplet formation in both wild-type and mutant p53; however, phosphorylation, DNA, and ATP can suppress the process and dissolve p53 droplets [517,609]. It has been proposed that LLPS droplet formation acts as a functional “on-off” switch for p53, where the compartmentalization of p53 into droplets prevents p53 transcriptional functions such as gene targeting and binding. Upon stress or PTM modification (phosphorylation) activation, p53 is released from the droplets to execute its physiological functions [609].

A recent study demonstrated that wild-type p53 expressed in living yeast not only had the capacity to form liquid-like, dynamic, unstable droplets that appeared and disappeared in response to the presence or absence of stress, respectively, but when overexpressed, was able to propagate into true amyloid-like prions that could seed other molecules, and at the same time suppressed p53 transcription activities to precipitate tumorigenesis [774], confirming earlier experimental observations where a seed of mutant p53C oligomers and fibrils accelerated wild-type p53C in a prion-like manner [770]. Both wild-type and mutant p53 proteins exhibited aggregation kinetics and morphology, closely resembling classical amyloidogenic proteins such as Aβ and α-syn, with mutants displaying enhanced amyloidogenicity and accelerated aggregation [775] which contribute to functional loss [770,773,776] and gain [777,778] associated with tumorigenesis [775,779].

In AD human brain tissues and animal models, the interaction between p53, pathological tau oligomers, and Aβ form aggregates resulting in the mislocalization and impairment of its important physiological functions in DNA repair [726,727,728]. Compared to healthy elderly controls, p53 in AD patients exhibited a 100% increase in p53 in the superior temporal gyrus, and induced the phosphorylation of tau in HEK293a cells in vitro [728]. Dysregulation of p53 such as unfolded p53 caused by oxidative stress [780] is a reliable biomarker for AD [781,782], whereas overexpression of the truncated p53 isoform p47 (Δ40p53 or p44) [783] in mice accelerated aging and increased tau fibrillation [782,784,785]. Tau was recently reported to have increased wild-type p53 expression post-translationally through the abnormal modification of MDM2, the E3 ubiquitin ligase which negatively regulates p53 [786,787,788,789]. Since the discovery of ubiquitin-positive aggregates in various neurodegenerative diseases, there has not been any clear consensus on the exact nature of the involvement of the ubiquitin–protease system (UPS) in neurodegenerative disorders [790].

#### 5.4.2. The Potential Regulation of Ubiquitination/SUMOylation in MLO Assembly and Dissolution by Melatonin in an ATP-Dependent Manner

The main function of UPS is to degrade and eliminate abnormal proteins damaged by oxidative stress and/or mutations after they are covalently bound to ubiquitin in an ATP-dependent pathway [791]. Many MLOs, such as stress granules formed as a result of RNA interactions, rely on ATP-dependent UPS-associated proteins such as ubiquitin complexed within their structures for proper assembly and disassembly [32,792,793,794,795]. Ubiquitin (Ub) is a highly conserved protein that targets proteins for degradation via covalent binding, and ubiquitination is an enzymatic cascade involving ubiquitin-activation (E1), ubiquitin-conjugation (E2), and ubiquitin-ligation (E3) which relies on ATP to provide energy to ultimately form an isopeptide bond between Ub and the targeted substrate [18,796]. LLPS can promote and enhance ubiquitination by providing a scaffolding of necessary proteins to accelerate ubiquitination processing, and aberrant LLPS may result in dysfunction of the UPS in neurodegenerative disorders [797]. Ubiquitin-positive protein aggregates have been identified in many neurodegenerative diseases [798]; therefore, it is believed that the failure to eliminate ubiquitinated proteins in the brain is one of the major causes of neurodegeneration [799]. Alternatively, it is possible that neurotoxicity arises from a deficiency of free Ub that could reduce proteasome activity rather than the accumulation of ubiquitinated aggregates often observed in neurodegenerative diseases [800]. Even though Ub can bind to Aβ peptides, interfering with clearance pathways, Ub bound non-covalently to Aβ has been observed to exhibit a lower tendency to aggregate, significantly reducing fibril formation and delaying amyloid fibril aggregation in a dose-dependent manner [801].

In eukaryotes, the UPS may be the most complex, extensive, cytosolic proteolytic enzyme system that performs essential functions [802] including cell growth and cycle control [803], apoptosis [804,805,806], inflammation [807,808], transcription [809,810], and signal transduction [811]. The UPS exerts a critical influence over protein quality control in neurodegeneration [812], mediating the degradation of more than 80% of normal and abnormal intracellular proteins in the human body [813,814]. The proteasome is the only known ATP- and ubiquitin-dependent protease in both eukaryotes and bacteria [815,816,817], and ubiquitin-related molecules have been reported to participate in the regulation of LLPS in the formation of MLOs [795,818,819]. The proteasome contains six distinct ATPase subunits that cooperatively coordinate substrate binding, deubiquitination, unfolding, and translocation [820]. The failure of a single mutated ATPase decreased the overall rate of ATP hydrolysis by 66%, and reduced the 2–3-fold ubiquitinated substrate stimulation of ATPase activity to zero [821]. Substrate degradation is directly linked and is proportional to ATP hydrolysis; therefore, it is not unreasonable to assume that ATP hydrolysis may be the rate-limiting step in UPS [821]. Thus, the maintenance of high cytosolic levels of ATP in the millimolar range by mitochondria [39,40] is not only requisite for the proper assembly and disassembly of MLOs; it is potentially indispensable for substrate degradation by UPS [821].

The theoretical maximum of ATP calculated from simultaneous measurements of extracellular acidification and oxygen consumption indicated that OXPHOS ATP production was close to or more than 16 times above glycolysis, at 31.45 ATP/glucose (maximum total yield 33.45) and 2 ATP/glucose, respectively [822]. Whether mitochondria can use OXPHOS to generate ATP is dictated by the fate of pyruvate upon glucose oxidation [823]. In mitochondria, pyruvate drives ATP production by OXPHOS and the TCA cycle via different enzymes. Pyruvate dehydrogenase complex (PDC) irreversibly converts pyruvate, NAD^+^, CoA into acetyl-coA, NADH and CO_2_. The phosphorylation of the E1α subunit of pyruvate dehydrogenase complex (PDC) by pyruvate dehydrogenase kinase 2 (PDK2) blocks the entrance of acetyl-coA into the tricarboxylic acid (TCA) cycle, inhibiting the OXPHOS production of higher ATP [824,825,826]. OXPHOS is believed to be the main initial energy production pathway used by neurons to fuel activities [827]; thus, alterations in PDK enzymes and/or their interactions with neurons and glial cell metabolism may affect the development of neurological disorders [828]. Decreased expression of PDC has been observed in post-mortem brain tissues from AD patients [829] as well as transgenic female AD mice [830].

VDAC is the gatekeeper that controls the export of ATP out of mitochondria into cytosol and the import of essential respiratory substrates such as ADP and Pi into mitochondria [395,399]; therefore, it is not surprising that VDAC has been demonstrated to be neuroprotective against Aβ-induced neuronal mortality [831] and essential for neurite maintenance and the prevention of demyelination after spinal cord injury [832]. The interactions between VDAC, APP, and Aβ in lipid rafts of neurons from the frontal and entorhinal cortex of human brains affected by AD showed enhanced dephosphorylation of the enzyme that correlated with cell death [833]. As discussed in Section 3.9, melatonin protects the functionality of the VDAC–CYB5R3 complex by reducing oxidative stress, lowering ROS that may induce lipid peroxidation, which can alter raft composition, thickness, curvature and elasticity [291] that may impact VDAC ion-channel opening/closure according to the force-from-lipid principle [382,383,384,385]. VDAC expressed in the plasma membranes of HT22 mouse hippocampal neuronal cells were quiescent under control conditions with normal ATP and an absence of apoptotic signals. Serum deprivation increased ROS and induced VDAC opening in the plasma membranes of hippocampal HT22 cells, resulting in mitochondrial dysfunction and increased apoptosis and autophagy. HT22 cells pre-loaded with 200 μM melatonin prior to serum deprivation did not exhibit VDAC activities. In the same manner, the addition of 4 mM ATP blocked the activation of VDAC channels [834]. The fact that both ATP and melatonin rescue neuronal cytotoxicity from VDAC-associated mitochondrial dysfunction may offer an explanation as to why p53 is found to be elevated in AD patients [728], and why tau increases wild-type p53 expression through the modulation of MDM2 [788,789,835], the E3 ubiquitin ligase which is also used by melatonin to activate p53 [836].

p53 is a transcription factor [519] that responds to a diverse range of stress signals [837] where it may promote survival and growth through the regulation of metabolic pathways [745], controlling protein synthesis and mRNA translation [838], and mediating energy metabolism under physiological and pathological conditions [839]. Mutant p53 has been reported to increase aerobic glycolysis and suppress mitochondrial OXPHOS, driving the “Warburg Effect” [840]. However, wild-type (WT) p53 has been reported to inhibit glycolysis and promote mitochondrial OXPHOS via the mediation of microRNA-34a and the IKK-NF-kappaB pathway [841,842,843]. WT p53 can regulate pyruvate metabolism in a manner that favors the conversion of pyruvate into acetyl-CoA, which then enters the TCA cycle that fuels ATP production in OXPHOS [844]. In irradiated mice, activation of wild-type p53 decreased PDK2 mRNA concentration in the colon and spleen, and increased the active, unphosphorylated form of PDC; by contrast, irradiated p53-null mice did not exhibit any decrease in PDK2 mRNA [844]. Active, unphosphorylated PDC converts pyruvate, NAD^+^, CoA into acetyl-coA, NADH and CO_2_, supporting the TCA cycle in OXPHOS to generate higher levels of ATP [824,825]. AD patients and transgenic AD mice show decreased PDC expression [829,830]; therefore, the regulation of PDK2/PDC by p53 may play an integral role in neuroprotection. Melatonin has been studied extensively for its ability to activate p53 [845,846,847] through phosphorylation [836,848,849,850,851,852], which is an ATP-dependent post-translational modification [365].

Breast cancer (MCF-7) and human colorectal carcinoma cells (HCT116) treated with 1 μM melatonin exhibited p53 activation and accumulation which inhibited proliferation and protected against DNA damage through the ATP-dependent phosphorylation of p53 at serine 15 (Ser15) [848]. Phosphorylation of p53 at Ser15 suppresses the inhibitive effect of MDM2 on p53 [853] and is also required for the maintenance of p53 physiological functions [854]. MDM2 is an E3 ubiquitin ligase that both inhibits the p53 transcription of target genes and acts as a molecular scaffold to promote p53 ubiquitination and the proteasome-dependent degradation of p53 [855]. Thus, MDM2 is often seen to be overexpressed in human tumors retaining wild-type p53 [856]. MCF-7 cells treated for 3 h with only 1 nM melatonin showed a dramatic overall cellular decrease in MDM2 content compared to control values [850]. The mechanisms involved that were observed [850] included the inhibition of Akt-PI3K-dependent MDM2 phosphorylation [851,857] together with the increase in p300 [858] via Sirt1 suppression [859]. In addition, a twofold increase in the concentration of ribosomal protein (RP) L11 was observed [850]. Aside from L11 [860,861], other RPs including S7 [862], L23 [863,864] and L5 [861,865] have also been reported to inhibit MDM2-mediated ubiquitination by binding to MDM2 to promote the activation and stabilization of p53 [866]. Intriguingly, the inhibition of ubiquitin was reported to cause a corresponding increase or decrease in SUMOylated proteins, with the implication that when the UPS cannot efficiently degrade targeted substrates, the proteins may be SUMO-modified and accumulate in MLOs [867]. Early studies have indicated the existence of an intricate interplay between SUMO and ubiquitin in response to genotoxic stress and DNA damage [868,869,870,871].

As part of the complex PTM/UPS modification system, SUMO modification, similarly to ubiquitination, is an ATP-dependent enzymatic cascade involving activating, conjugating, and ligating E1, E2, and E3 enzymes, respectively [872,873]. Small ubiquitin-like modifiers (SUMO) recognize and conjugate many protein substrates that may also be targeted by ubiquitin [874,875,876], but often with different effects [877]. The SUMOylation of proteins can alter interaction properties that may change subcellular localization, function and stability [878,879]. SUMOylation mediates the intranuclear and nucleo-cytoplasmic translocation of proteins regulating circadian rhythm [880], neuronal and synaptic functions [881,882,883], apoptosis [884], and protein degradation [875,885]. Unlike ubiquitin, SUMO-binding proteins involve covalent [886] as well as non-covalent [887] interactions that are believed to exert great influence over nuclear processes such as transcription, replication, and the maintenance of genomic integrity [888]. Embryos of mice bred without the SUMO-conjugating enzyme E2 did not survive beyond the early postimplantation stage [889]. The interplay between ubiquitin and SUMO in the nucleolar compartment may be driven by LLPS [890,891], because the inhibition of ubiquitination leads to an accumulation of SUMOylated proteins that condense into MLOs known as promyelocytic leukemia proteins (PMLs) in nucleoli [867,892].

Nucleoli PMLs are phase-separated quality control MLOs that compartmentalize misfolded proteins for clearance [893]. In mammalian cells, defective ribosomal products [894] may misfold as a result of DNA mutations [895] and damage to mRNA responsible for transcription and/or translation during protein synthesis [896,897,898,899]. The defective clearance of misfolded proteins often aggregates into protein structures in either an amyloid or amorphous state. Amyloid aggregates are insoluble, structured, higher-order assemblies [900,901,902], whereas amorphous aggregates are disordered and may contain soluble proteins [903]. Many neurodegenerative diseases, including Alzheimer’s, Parkinson’s, and Huntington’s, are associated with amyloid aggregates [904]. Failure of SUMOylation may result in the ineffective clearance of defective proteins that affect neurodegenerative disorders [905,906]. It is believed that most familial PD is caused by mutations in parkin, a ubiquitin E3 ligase that regulates the turnover of RanBP2, the SUMO E3 ligase, by catalyzing its ubiquitination to promote proteasome degradation and clearance [907].

In the cytoplasm, exposure to heat [908,909], oxidative stress [910], and osmotic stress [819,911] can cause the misfolding of proteins often associated with neurodegenerative disorders. Cells respond to various stress factors by forming cytoplasmic stress granules which are dynamic MLOs that can conserve energy and limit protein synthesis by transiently sequestering ribonucleoproteins (RNPs) such as non-translating mRNAs and RNA-binding proteins to downregulate bulk translation. Upon the removal of stress conditions, these dynamic SG MLOs in the cytoplasm are disassembled, releasing stored RNPs to reassume protein synthesis [541,542,912,913]. SG components include RBPs such as TDP-43, FUS, tau, and p53; therefore, mutations and aberrant SG dynamics may significantly contribute to neurodegenerative disorders [540,837,914]. Both ubiquitination and SUMOylation have been reported to regulate SG dynamics, where the ubiquitination–protease system [915] and SUMO-primed ubiquitination facilitate the timely resolution and disassembly of SG upon stress release, preventing aberrant SGs that may result in disease-linked pathological aggregates [915,916]. Consequently, failure to SUMOylate eIF4A2 (or DDX2B), a DEAD-box RNA helicase that acts as a scaffolding protein, impairs stress granule formation [917]. Melatonin has been reported to induce and enhance SUMOylation for the effective degradation of Aβ in AD mice models [918].

Frontal cortex tissues of double-transgenic APP/PS1 AD mice that were given daily intraperitoneal injections of melatonin at pharmacological concentration of ~10 mg/kg (0.3 mL, 10 µg/µL) for 3 weeks exhibited a significant degradation of Aβ as a result of the SUMOylation of the amyloid precursor protein (APP) intracellular domain (AICD) at lysine 43 by the SUMO E3 ligase protein inhibitor of activated STAT1 (PIAS1) [918]. AICD SUMOylation not only enhanced the clearance of Aβ and amyloid plaque in vivo [918], but the covalent SUMO-modification of amyloid precursor protein (APP) at lysines 587 and 595 by SUMO E2 ligase has also been observed to reduce Aβ aggregates in vitro [919]. Compared to wild-type AICD, SUMOylated AICD was actually more effective in the reduction in Aβ levels and the suppression of amyloid plaque accumulation [918]. APP/PS1 double-transgenic AD mice treated with melatonin exhibited an enhanced expression of AICD accompanied by marked improvements in both spatial learning and memory deficits, possibly due to the induction of AICD SUMOylation by melatonin [918].

Stress granules are formed in response to external stress factors; therefore, hypoxia, heat, oxidative, osmotic, and genotoxic stress can also significantly increase SUMO conjugate levels as a protective response [920]. Under severe oxygen and glucose deprivation, overexpression of SUMO-1 or SUMO-2 in human neuroblastoma SHSY5Y cells increased survival and ischemic tolerance [921]. SUMO conjugation may be correlated to intracellular ROS levels in a dose-dependent manner. In HeLa cells, high levels of H_2_O_2_ increased SUMO conjugation, but exposure to low levels of H_2_O_2_ (1 mM) induced a severe, rapid deSUMOylation within 5 min, resulting in the disappearance of SUMO conjugates including transcription factors [922]. Even though SUMOylation may exert neuroprotective functions [923], a dysregulated SUMO system can also negatively impact Aβ and tau aggregates in AD [924] due to the fact that SUMOylation not only controls protein–protein interactions [886] but also regulates the transcriptional control of RNA through various post-transcriptional modifications [925].

### 5.5. Post-Transcriptional Modifications of RNA by m^6^A Regulate Phase-Separated MLOs

Ribonucleic acid (RNA) is a single-stranded molecule with alternating ribose and phosphate groups attached to adenine, uracil, cytosine or guanine bases. The evolution of RNA is believed to precede that of DNA; nonetheless, the origin of the “RNA World” has not been resolved to date [926,927,928]. Perhaps due to its earlier evolution, RNA controls gene regulation at multiple levels [929,930,931]. Gene expression is essentially the transfer of genetic information from deoxyribonucleic acid (DNA) to proteins by RNA using both coding messenger RNA [932,933] and non-coding RNA [934,935,936,937]. Abnormal gene transcription may alter gene expression which often results in neurodegenerative diseases. During healthy aging, changes in the expression of key switch genes in the brain may cause neurodegenerative disorders [938]. Post-transcriptional RNA modifications are believed to have important roles in gene expression and regulation [939,940].

Phase-separated MLOs are enriched in RNA and RNA-binding proteins with IDRs [527,630,941]. RNA can be considered as an architectural element that not only seeds the nucleation of condensates but affects the size and composition of condensate phases [23]. Both the transcriptional regulation and post-transcriptional regulation of genes are now believed to be directly associated with phase separation [942]. Activation domains of transcription factors undergo phase separation to facilitate gene activation [109]. Henninger and colleagues (2021) revealed that at gene transcription sites, optimal condensate formation and transcription are co-dependent, where low levels of RNA enhanced condensate formation, supporting transcription; however, high levels of RNA dissolved condensates, terminating transcription. Control of condensate formation and dissolution during transcription processes are dependent upon fluctuations in RNA abundance that alter the electrostatic charge balance in condensates that contain transcription factors [525].

Transcription factors (TFs) can undergo LLPS to form dynamic regions that compartmentalize and concentrate other TFs, enriching transcription-related proteins to activate the transcription of target genes [527]. RNA properties such as composition, length, structure, modification, and expression level can modulate the size, shape, viscosity, liquidity, surface tension, and composition of these condensates [529,943,944]. Experimental studies have showed that longer, more structured RNA prevents the aggregation of the p53 DNA-binding core domain (p53C) in vitro [567]; therefore, it has been proposed that the larger surface area and charge of structured RNAs could potentially act as globular nanoparticles that induce changes in bound proteins to initiate fibrillation [945] or suppress aggregation [946].

Aberrant RNA–RNA interactions leading to the sequestration and/or dysregulation of RNA-binding proteins in MLOs may be one of the major driving forces behind neurodegenerative diseases [11,542,947,948,949]. Aberrant LLPS as a result of deficient RNA-binding in TDP-43 can form pathogenic, insoluble aggregates that are excluded from physiological SGs. On the other hand, with RNA-binding, TDP-43 phase-separated into dynamic inclusions that were recruited into RNA-rich, fluid compartments within SGs [28]. The formation of SGs in response to various stress conditions, including oxidative stress, involves the sequestration of translationally stalled mRNA and RNA-binding proteins to conserve energy and downregulate bulk translation [541,542,912,913].

The mechanism of selection for mRNA inclusion in SGs is determined by mRNA modification mediated by a prevalent methylation at position 6 of adenosine (m^6^A) in the 5′ UTRs of mRNA [950]. m^6^A mRNA modification is dynamic, reversible, and has been observed to be oxidative-stress-dependent. Stress-induced methylation is recognized by the m^6^A cytoplasmic “reader” protein, YTH domain family 3 (YTHDF3) [951], which then relocates the selected mRNA transcripts into SGs [950]. The ability of m^6^A to affect heterogeneous RNA and protein contents of SGs resulting from stress-specific differentiation in composition, dynamics of assembly and disassembly may ultimately determine the viability or pathology of cells in neurodegenerative diseases [540].

#### RNA Regulation by N^6^-Methyladenosine (m^6^A) in Neurodegenerative Disorders

Modification of eukaryotic messenger RNAs (mRNAs) occurs mostly via N^6^-methyladenosine (m^6^A), which involves the transfer of a methyl group to the sixth position of the purine ring in RNA adenosine [952]. m^6^A is installed by m^6^A methyltransferases and removed by m^6^A demethylases. RNA splicing, transcription, stability, and metabolism are all regulated by RNA m^6^A modifications [953,954,955,956]. m^6^A mediates structural switches that affect RNA stability and activity, regulating the access of RNA-binding proteins to their RNA binding motifs [957]. Among hundreds of types of RNA modifications identified [958], m^6^A is possibly the most prevalent internal, dynamic, reversible chemical modification identified to date, and plays critical roles in the growth, differentiation, and metabolism of cells [952,959]. An evolutionarily conserved RNA modification, m^6^A RNA methylation is involved in most aspects of RNA processing that may affect the regulation of cellular processes [960] such as immune modulation [961,962,963], fat metabolism [964], circadian rhythm [965], fertility [966,967,968,969], and brain plasticity and development [970].

The m^6^A RNA modification of eukaryotic RNAs is dynamic and reversible, where the methylation of mRNAs, tRNAs, rRNAs, and long non-coding RNAs by “writers” (RNA methyltransferases) such as METTL3 [971] is removed by “erasers” (RNA demethylases) such as FTO and ALKBH5 [972,973], and recognized by “readers” (m^6^A-binding proteins) such as YTH domain proteins [974,975]. Dysregulations of these m^6^A “writers”, “readers”, and “erasers” are increasingly associated with degenerative and metabolic diseases. Fat mass and obesity-associated (FTO) protein, the m^6^A “eraser” associated with human obesity and energy homeostasis [976,977,978,979], was found to be upregulated in breast cancer [980], hepatocellular carcinoma [981], melanoma [982], and acute myeloid leukemia (AML) [983]; however, the downregulation of FTO in vivo and in vitro enhanced invasion and metastasis in epithelial cancers [984]. ALKBH5, another m^6^A “eraser”, was found to be overexpressed in the tumorigenesis of glioblastoma stem-like cells [985]. The METTL3 m^6^A “writer” was also identified as a critical regulator of a chromatin-based pathway that maintained cells in a leukemic state, where the inhibition of METTL3 removed the myeloid differentiation block in human and mouse AML cells [986]. Depletion of YTHDF1, the m^6^A “reader”, was able to enhance antitumor immune responses in the dendritic cells of tumor-bearing mice [987]. The dysregulation of m^6^A is increasingly associated with tumorigenesis [955,959,988,989] and neurodegenerative disorders [990,991,992,993].

m^6^A methylation may be highest in the brain, regulating embryonic stem cell differentiation and brain development [992,994,995,996,997,998,999]; however, dysregulated m^6^A methylation potentially drives neurodevelopmental disorders [993]. Investigations employing high-throughput sequencing comparing m^6^A RNA methylation in the brains of double-transgenic APP/PS1 with those of control mice revealed statistically significant elevations of m^6^A methyltransferase METTL3 and downregulations of m^6^A demethylase FTO in the cortex and hippocampus of AD mice [991]. Post-mortem human AD brain samples showed distinct aberrant expression of m^6^A methyltransferases where METTL3 and the RNA-binding motif protein 15B (RBM15B) were downregulated and upregulated in the hippocampus, respectively. METTL3 was observed to be accumulated in the insoluble fractions of tau proteins, possibly implying an epitranscriptomic mechanism in altered gene expression in neurodegenerative disorders [990]. RNA epitranscriptomics regulation [1000] may provide additional speed and specificity [1001] to facilitate the transcriptional regulation of gene expression by epigenetic mechanisms [1002].

Dysregulation of m^6^A modifiers can lead to changes in the regulation of gene expression, affecting cancer [1003,1004], neurodegenerative diseases [990,991,1005], aortic dissection [1006], blood pressure regulation [1007], and cardiac function [1008]. More than 50% of all human cancers have been associated with missense [758] and/or synonymous mutations of the p53 gene [759,760,761]. An analysis of datasets from the Cancer Genome Atlas Research Network (TCGA) acute myeloid leukemia (AML) study revealed that mutations and/or copy number variations in genes that write, read, or erase m^6^A methylations, such as METTL3, METTL14, YTHDF1, YTHDF2, FTO, and ALKBH5, are significantly correlated with p53 mutations in AML patients [1009]. Most (93.6%) AML patients with mutated p53 have ≥1 genetic alteration(s) of these m^6^A regulatory genes. In addition, their overall and event-free survival is worse than patients without m^6^A genetic alterations [1009]. The loss of the m^6^A methyltransferase METTL3 in hepatocellular carcinoma cells (HepG2) caused alterations in gene expression and alternative splicing in more than 20 genes, including MDM2, MDM4, and p21, involved in the signaling of p53 [1010]. R273H is a hot-spot missense mutation in the p53 gene [1011,1012] which can promote cellular malignancy [1013], migration and metastasis [1014]. m^6^A methylation by METTL3 at the point-mutated codon 273 (G > A) of p53 pre-mRNA promoted a preferential pre-mRNA splicing that produced p53 R273H mutant genes that were resistant to multiple anticancer drugs in colon cancer cells [1015]. However, silencing METTL3 expression or inhibiting RNA methylation substantially increased the level of phosphorylated p53 protein (Ser15), allowing cells with heterozygous R273H mutations to respond normally to anticancer drugs [1015].

Aberrant RNA–RNA interactions leading to the sequestration and/or dysregulation of RNA-binding proteins in MLOs may be one of the major driving forces behind neurodegenerative diseases [11,542,947,948,949]. Section 5.5 discussed the formation of SGs in response to various stress conditions involving the sequestration of translationally stalled mRNA and RNA-binding proteins [541,542,912,913], where the mechanism of selection for mRNA inclusion in SGs is determined by oxidative stress-dependent m^6^A mRNA modifications by “reader” YTHDF3 [950,951]. The formation of MLOs such as SGs and P-bodies [63] enriched in translationally stalled mRNAs is dependent upon m^6^A-binding protein YTHDF. Depletion of YTHDF1/3 in human bone osteosarcoma epithelial cells (U-2 OS) inhibited SG formation and the recruitment of mRNAs into SGs [1016]. YTH proteins themselves undergo LLPS, and must bind to m^6^A-RNA before they can be complexed into stress granules, implying that polymethylated m^6^A-RNA may act as a scaffold for YTH proteins, causing them to undergo LLPS through interactions within their own low-complexity domains [1017,1018]. Therefore, m^6^A can be regarded as a beacon that attracts YTH proteins into stress granules [1019]. PTMs such as ubiquitination and SUMOylation have been reported to regulate SG dynamics, where the ubiquitination–protease system [915] and SUMO-primed ubiquitination facilitates the timely resolution and disassembly of SG upon stress release [915,916]. Melatonin has been reported to enhance AICD SUMOylation in APP/PS1 AD mice, improving spatial learning and memory deficits; therefore, melatonin may regulate biomolecular condensates via RNA and RNA m^6^A modifications.

### 5.6. Potential Regulation of RNA and RNA m^6^A Modifications by Melatonin

Even though AICD SUMOylation has been shown to exert beneficial effects in transgenic AD mice models, the study of SUMOylation in neurodegenerative disorders and other diseases has led to controversial and often contradictory observations, because pathways can undergo SUMOylation at different sites, yielding conflicting consequences. For example, the SUMOylation of many important proteins in AD, including APP and tau, have been associated with the pathogenesis of AD [1020,1021] and PD [1022]. On the other hand, SUMOylated misfolded proteins are targeted for ubiquitination by ubiquitin ligase RNF4, then subsequently degraded by UPS [1023]. In vitro experiments have reported that the overexpression of SUMO-1 and SUMO E2 enzyme ubc9 decreased Aβ aggregates [919], and only 10% SUMOylated α-synuclein was enough to prevent aggregation [1024]. Interestingly, fibroblasts exposed to staurosporine-induced oxidative stress exhibited reduced apoptosis as a result of α-synuclein aggregates promoted by SUMOylation [1025]. SUMOylation is essentially a stress-responsive PTM which is rapidly increased upon cellular stress to reprogram cells [1026,1027,1028] and mitochondria [1029] for survival. SUMO is also known for its inhibition of transcription factors [1030]. Upon stimulation by oxidative stress, transcription factor E2F1 was efficiently SUMOylated to initiate cell cycle arrest to increase survival [1031]. Therefore, the association of SUMO with proteins implicated in neurodegenerative disorders which are also transcription factors such as SOD1 [1032], p53 [519], tau [520], TDP-43 [521], and FUS [523] would not be unexpected, although the occasional negative outcomes may require further elucidation [1033,1034,1035,1036,1037,1038]. It is therefore not surprising to find that the SUMOylation of m^6^A “writers” and “readers” is also associated with the progression of cancer.

The SUMOylation of METTL3 was found to promote tumor growth and colony formation in human non-small cell lung carcinoma (NSCLC) cells (H1299) [1039], where the SUMO1 modification of METTL3 at lysine residues K177, K211, K212, and K215 dramatically repressed methylation that decreased mRNA m^6^A levels in vitro and in vivo—a process that can be reduced by deSUMOylation enzyme sentrin/SUMO-specific protease 1 (SENP1) [1039,1040]. In a study of liver cancer, the SUMO1 modification of METTL3 promoted tumor progression with high metastatic potential [1041]. However, in lung cancer, the SUMOylation of m^6^A “reader” YTHDF2 by SUMO1 at lysine residue K571 in vitro and in vivo increased the binding affinity of YTHDF2 to m^6^A-modified mRNAs, altering gene expression profiles, resulting in the increased proliferation, migration, colony formation and tumor growth of lung cancer H1299 cells [1042].

The YTHDF2 m^6^A “reader” targets and destabilizes m^6^A-modified mRNAs, facilitating the localization and degradation of m^6^A mRNA in MLOs such as P-bodies [63,953]. Ultimately, the amount of RNA released into cytoplasm could be the factor that determines the assembly and disassembly of MLOs, where a low level of negatively charged RNA could interact with positively charged proteins to promote phase separation and the formation of condensates, whereas high levels have the opposite effect in repelling proteins with a positive charge to dissolve condensates [525]. The promotion of an mRNA nuclear export is controlled by m^5^C, a ubiquitous post-translational RNA modification found in mRNAs [1043]. YTHDF2 has been observed to bind directly to m^5^C in RNA, significantly regulating 208 out of 1350 identified m^5^C sites that may affect pre-rRNA processing through the modification of m^5^C levels in rRNA [1044]. More importantly, melatonin has also been found to modulate YTHDF2 as well as METTL3.

Stimulation of oncogene Ras led to the suppression of YTHDF2 that stabilized the transcription of MAP2K4 and MAP4K4, upregulating the senescence-associated secretory phenotype (SASP) in human ovarian surface epithelial cells (HOSEpiC). Treating HOSEpiC cells with 1 μM of melatonin enhanced the expression of YTHDF2, reversed telomere shortening, and blocked Ras-induced growth arrest [1045]. The activation of cytoplasmic YTH domain “readers” [951] has been inversely correlated with oxidative stress. Upon the induction of oxidative stress, YTHDF1 increases localization to SGs to lower the activation energy barrier and reduce the critical size for SG condensate formation [1016]. Substituting 1 μM melatonin with 10 mM *N*-acetyl-l-cysteine (NAC), an antioxidant, also augments YTHDF2 expression in oxidative-stress-induced senescent cells; therefore, it is believed that oxidative pathways may negatively regulate YTHDF2 expression [1045]. On the other hand, the mechanism(s) involved in the modulation of METTL3 by melatonin may not be as straightforward.

Adult male C57BL/6J mice pretreated with melatonin intraperitoneal injections (25 mg/kg b.w./day × 14 days) exhibited attenuated cell viability loss, ROS generation, mitochondrial dynamics imbalance, and mitophagy in spermatogonial stem cells (SSCs) induced by daily intraperitoneal injections with chromium (VI) (16.2 mg/kg b.w./day × 14 days), an environmental toxin and carcinogen that can cause male infertility by damaging SSCs [1046,1047,1048]. In vitro mouse SSCs/progenitor cells treated with 10 μM chromium (VI) exhibited decreased METTL3 mRNA levels, but cells pretreated with 50 μM melatonin were able to attenuate the downregulation of METTL3 [1046]. An interesting observation was the significant elevation of METTL3 to levels above controls in the melatonin-only samples, whereas YTHDF2 levels were significantly elevated above the control samples in the melatonin + chromium (VI) cells after 4 h, which again supports the theory that the expression of YTHDF2 may be correlated with stress levels [1016], and can be increased by the presence of melatonin, and perhaps other antioxidants [1045].

On the other hand, melatonin was found to decrease METTL3 expression and modification in another report. m^6^A regulates the pluripotency of embryonic stem cells (ESCs) [1049]. Treatment with 10 μM melatonin maintained the stemness features of ESCs for more than 90 days (45 passages) via the marked suppression of global m^6^A modification and significant reduction in m^6^A “writer” METTL3 [1050]. Melatonin treatment decreased m^6^A mRNA methylation and altered the subcellular location of METTL3, preventing m^6^A-dependent mRNA decay to stabilize key pluripotency factors Nanog, Sox2, Klf4, and c-Myc [1050], known to be destabilized by m^6^A methylation [1051]. It has been proposed that melatonin could decrease METTL3, increasing ESC pluripotency via the MT1-JAK2/STAT3-Zfp217 signal axis. Zinc finger protein 217 (ZFP217) has been reported to activate pluripotency genes and sequester METTL3 [1052]. Using doxycycline to induce an 85% knockdown of ZFP217 in ESCs treated with melatonin, or the depletion of melatonin receptor 1 (MT1), failed to produce similar effects of m^6^A modification compared to wild-type ESCs treated with melatonin [1050]. However, if knocking down ZFP217 (reduction in ATP by 25% in prostate cancer cells [1053]) and/or using doxycycline (~80% reduction in ATP in hypoxic stem-like prostate cancer cells [1054]) lowered ATP production in ESCs used in the experiment [1050], then it is possible that even in the presence of melatonin, the lack of ATP led to the failure of DDX3, an ATP-dependent helicase that regulates m^6^A mRNA methylation.

m^6^A mRNA methylation can be oxidatively reversed or “erased” by m^6^A demethylases such as FTO [976,1055] and ALKBH5 [973,1056]. Just as the suppression of METTL3 enhances ESC pluripotency [1050], the deficiency of “erasers” such as ALKBH5 is associated with testicular dysfunction, resulting in compromised spermatogenesis [1056]. In addition, the aberrant overexpression of ALKBH5 in AML enhances the self-renewal of leukemia stem/initiating cells, often resulting in poor prognosis in AML patients [1057]. In fact, the demethylation of mRNAs by ALKBH5 in stem cells is mediated by DEAD-Box Helicase 3 (DDX3 or DDX3X), an ATP-dependent RNA helicase. DDX3 was found to modulate the demethylation of mRNAs via interactions between the DDX3 ATP domain and the DSBH domain of ALKBH5 [1058]. DDX3 is expressed in adult germ cells, whereas the expression of DDX3 in embryonic stem cells is the highest during early development [1059]. DDX3 was found to be overexpressed in undifferentiated pluripotent stem cells, compared to differentiated cells, and the abrogation of DDX3 expression in multiple stem cells resulted in reduced proliferation but increased differentiation, while at the same time, lowered potency to induce teratoma formation [1059].

NLRP3 inflammasome, a widely documented target of melatonin associated with pathological protein aggregates in neurodegenerative disorders [311,312,313,314], is a stress-induced supramolecular complex formed by phase separation [269,270,271] (Section 3.6). DDX3 is the determining factor that could favor the transition of NLRP3 into pro-death, stable, prionoid-like complexes containing self-oligomerizing specks that cannot be easily disassembled once they are formed [304,305] over the formation of reversible, pro-survival stress granules [304,310] (Figure 2). Melatonin has been widely reported to inhibit NLRP3 inflammasome inactivation; therefore, the connection between melatonin, DDX3, and other ATP-dependent RNA helicases may simply originate from the two most basic but quintessential elements that have been shaping and defining MLOs since the very beginning of life—ATP and RNA.

### 5.7. The Ancient Relationships between Melatonin, ATP, RNA, and Membraneless Organelles

When life originated, LLPS driven by multivalent macromolecular interactions might have been the organizing principle behind the subcellular compartmentalization of MLOS in eukaryotes and prokaryotes [2,82,277,428]. The assembly and disassembly of dynamic, transient MLOs containing RNAs, nucleic acids, and proteins [1] is tightly correlated with ATP. DEAD-box (DDX) proteins are RNA-binding ATPases that couple cycles of ATP binding and hydrolysis to changes in affinity for single-stranded RNA [1060,1061], where ATP-bound DDXs exhibit a tight affinity for RNA [1062]. DDX is involved in all aspects of RNA metabolism, from translation initiation, pre-mRNA splicing, mRNA export and decay, and ribosome biogenesis [1063]. DDX can promote RNA–protein complex remodeling, RNA duplex unwinding, and duplex annealing [1061,1063] (Figure 1).

Adenosine triphosphate (ATP) is one of the four nucleotide monomers used during RNA synthesis [1064]. RNA has been demonstrated to bind to ATP with high affinity and specificity [1065]. The tight relationship between ATP and RNA may date as far back as the “RNA world”, when ATP existed as an important cofactor of a metabolic system composed of nucleic acid enzymes prior to the evolution of ribosomal protein synthesis [1066,1067]. The addition of an unstable, third phosphate onto adenosine diphosphate (ADP) produces ATP. The transfer of the third phosphate released during hydrolysis drives energetically unfavorable but essential metabolic reactions in living organisms [1067,1068]. When RNA substrates are engaged during RNA rearrangement and unwinding processes, DEAD-box RNA helicases can display different open or closed conformations when bound to ADP or ATP, respectively [1069,1070,1071]. DDXs have also been reported to form stable, persistent complexes with RNA during RNA clamping [1072].

Cells rely upon RNA to regulate condensates because RNA molecules contain powerful electrostatic forces due to the high negative charge densities buried in their phosphate backbones [530,531,532]. Therefore, a low level of RNA with negative charge could interact with positively charged proteins to promote phase separation and the formation of transcriptional condensates, whereas high levels of negatively charged RNA could repel proteins with positive charges to dissolve condensates [525]. In the regulation of MLO assembly and disassembly dynamics, DDXs such as DDX3, DDX4, and DDX6 may function as molecular switches that direct mRNA into RNA granules such as P-bodies and stress granules for transient storage or decay, as well as the timely, necessary resolution and disassembly of these granules [49,1073,1074,1075,1076,1077]. It is important to note that the export of nuclear mRNA into cytoplasm is regulated by DDX19, an ATP-dependent RNA helicase with many important functions [1078].

Since its discovery in *Saccharomyces cerevisiae* in 1999, DX19 (human)/Dbp5 (yeast) [1079] has been associated with important functions involving mRNA export and remodeling [1062,1079,1080,1081], mRNA expression [1082], and translation [1076], as well as DNA transcription and metabolism [1083,1084]. One of the most important functions of DDX19 in the context of MLO dynamics is the export of nuclear mRNA via nuclear pore complexes (NPCs). NPCs are huge, highly conserved, macromolecular structures comprising ~1000 protein subunits (nucleoporins) that perforate the nuclear envelope, fusing inner and outer nuclear membranes to create pores as well as a passive diffusion barrier of disordered proteins [1085]. NPCs not only mediate mRNA export into the cytosol and bidirectional protein transport, but they may also be transcription regulators which are spatial organizers of the genome due to their ability to interact with chromosomal loci to promote transcriptional activation, repression, and poising [1086].

It has been proposed that the ATP-dependent catalytic cycle of DDX19 involves the cycling between open and closed conformations to bind RNA for export into cytoplasm [1085]. Even though mutant Dbp5/DDX19 which could not bind RNA are unable to export mRNA in both yeast and human cells [1080], ATP binding and hydrolysis are also necessary for Dbp5/DDX19 to engage nuclear pore complexes for the optimal transport of mRNA into cytoplasm [1085]. DDXs have been shown to regulate the formation of phase-separated condensates such as stress granules and P-bodies in vivo and in vitro [69]; therefore, the overexpression of DDX19 may actually prevent the formation of SGs, as reported by an experimental study showing that the overexpression of DDX eIF4A [1087] together with ATP prevented drug-induced RNA condensate formation in vitro [1088]. The nuclear export of mRNA by DDX19 is reliant upon functional NPCs; therefore, the relationship between RNA and lipid domains in nuclear envelopes presents a deeper perspective into the role of melatonin in MLO dynamics.

NPCs can be visualized as thousands of toroid-shaped “ultradonut”-like pores with extremely high curvatures generated by nanoscale buckling instabilities triggered by membrane stresses during nuclei growth [1089]. These “ultradonuts” fuse the outer (ONM) and inner (INM) nuclear envelope (NE) membranes, which are lipid bilayers [1090,1091]. The NE ONM faces the cytoplasm and is a continuation of the ER [1092,1093]. It has been proposed that the ER is the source of the membrane for NE assembly [1094]. The fact that ER membranes are enriched with membrane-associated mRNAs and RNAs [71,72,73] may explain why MLOs such as P-bodies are formed at close proximity to ER membranes [70], because the assembly of MLOs such as P-bodies are dependent upon mRNAs and RNAs [67]. The composition of lipids in the NE is dominated by phosphatidylcholine with extremely high levels of negatively charged lipids and cholesterol, and reconstituted nuclear membrane vesicles have been seen to be more ordered than classical POPC membranes [1095]. Compared to classical plasma membranes, human nuclear envelopes are at least two orders of magnitude more elastic, with exceptionally high fluidity to stabilize large, dynamic, deep-penetrating invaginations that deform the membranes. The functions of these invaginations are as-yet unclear, although appear to be involved in calcium signaling and gene expression [1095]. Morphological changes to NEs due to the dysregulation of membrane lipid composition may lead to pathological outcomes [1095,1096].

As early as 1979, the regulation of nuclear RNA release was found to be directly correlated with nuclear membrane fluidity where a reduction in membrane fluidity caused a linear decrease in RNA release [1097]. It has been proposed that NPC biogenesis may be dependent upon the fluidity of NE membranes [1098]. Cells of *Saccharomyces cerevisiae* with defects in regulating membrane fluidity assembled NPCs that were defective, whereas the restoration of membrane fluidity via the addition of membrane-fluidizing agents attenuated defects in NPC biogenesis and normalized mRNA export [1099]. The peroxidation of lipids in NE [1100,1101] may reduce membrane fluidity. Lipid peroxidation can alter molecular structures, creating amphiphilic subpopulations and leading to significant changes in the phase behavior of lipid membranes that can affect the integrity and fluidity of membranes [214,318,343,344,353,354,355]. The preferential location of melatonin in bilayer lipid headgroups enables dynamic interactions that reduce bilayer thickness and increase bilayer fluidity [338,341,356]. The presence of both hydrophilic and lipophilic moieties in melatonin also facilitates the scavenging of both aqueous and lipophilic free radicals [411], especially ^•^OH [448] and ^•^OOH, the two most prevalent ROS responsible for the chain oxidation of unsaturated phospholipids [465,466] such as phosphatidylcholine, the dominant lipid in NE [1095] (Figure 1).

Membranes of NE must be tightly curved to support NPCs [1102]. Lipid components of the nuclear pore membrane may promote membrane curvature, maintaining a convex (positive) curvature along the surface of the membrane connecting the outer and inner membranes, and a concave (negative) curvature in the central plane of the pore membrane [1103,1104]. Despite the fact that many NPC proteins have been proposed to induce and/or stabilize membrane curvature by amphipathic helix insertion into the lipid bilayer [1105,1106], key questions on how NPCs promote membrane curvature remain unresolved [1107]. It is possible that nuclear lipid domains play an important role in the generation and stabilization of membrane curvature and fluidity in NE, because membranes themselves can affect local protein concentrations [360] where high curvature lipids that form rafts may attract specific proteins that can further enhance membrane curvature [361,362,363,364]. Adsorption of proteins onto membranes can modulate composition of the lipid bilayers because lipids may potentially flow to accommodate changes in membrane curvature during protein adsorption. These changes result in alterations to membrane tension that reflect the residual local tension that adjusts the difference between the actual mean curvature and the imposed spontaneous curvature [1108].

During protein membrane adsorption, the complex interactions between lateral membrane organization and proteins often enhance the propensity of membrane lipids to form domains or to phase-separate [1109]. These domains may, in turn, act as anchors for the adsorbed proteins [116]. The formation of nuclear lipid microdomains is especially relevant because NPCs are believed to be transcription regulators [1086]. An in vivo experiment using Sprague Dawley female rats discovered the existence of nuclear lipid raft microdomains that acted as platforms for transcription processes during RNA synthesis. Compared to sham-operated animals, lipid microdomains isolated from nuclei exhibited a lipid composition that was associated with DNA replication and transcription during cellular proliferation in liver regeneration, and these nuclear raft domains were especially enriched in labeled uridine when there was increased RNA synthesis [1110]. RNA and phospholipids may have a long-standing relationship; the two molecules have been shown to form heteromeric weak bonds that could regulate membrane permeability [1111,1112]. Human tRNASec was demonstrated to show increased binding affinity for lipid rafts [1113], and free RNA 10 molecules would preferentially associate with L_o_ lipid raft domains at 18 °C with ~80% binding, whereas increasing the temperature to 23 °C lowered the binding affinity to ~58% due to a corresponding increase in the non-raft L_d_ phase that discouraged binding [1114]. Cells may use melatonin to control temperature fluctuations that could affect RNA binding affinities. Melatonin stabilized lipid L_o_–L_d_ phase separation over a range of temperatures (tested up to 45 °C), preserving nanoscopic lipid domain structure and composition, possibly by reducing membrane line tension [350]. Lipid peroxides often induced nanometer-scale rafts to grow to micron sizes, accompanied by increased line tension in the order of several piconewtons [206,218,296]. As a potent antioxidant, melatonin may also be used by organisms to preserve membrane tension and fluidity, and stabilize L_o_-phase lipid rafts in cells and nuclei (Figure 1).

The relationship between melatonin and RNA is likely an ancient one that might date as far back as ~4 billion years ago, possibly after the height of the “RNA world” [1115,1116,1117], when a proposed gene duplication event at ~3.5 Ga involving CP43 and CP47, enzymes unique to photosystem II (PSII), marked the beginning of water oxidation [431]. Regulation of the synthesis and degradation of the evolutionarily conserved PSII D1 reaction center is mediated by post-translational RNA modulations [1118,1119,1120] and the presence of ATP [1121] in a light-dependent manner, where synthesis and/or degradation is induced by light but ceased in the dark. Unlike animals [1122], melatonin in plants is increased by the presence of light [1123,1124], and treatment with melatonin enhanced the synthesis of PSII D1 reaction centers in tomato seedlings under salt stress [1125]. Cyanobacteria, the only known prokaryote capable of water oxidation [431] which also produces melatonin [421,422], has recently been shown to exhibit circadian rhythm in the formation and dissolution of MLOs that remained soluble during daylight, but became reversible, insoluble condensates at night in an ATP-dependent manner [432]; therefore, it is not unreasonable to hypothesize that the relationship between melatonin, MLOs, ATP, and RNA was already in existence at ~3.5 Ga. The presence of melatonin in primitive unicellular organisms including *Rhodospirillum rubrum* and cyanobacteria, precursors to mitochondria and chloroplasts, respectively [415,423,424,425], may have conferred protection against endogenous and exogenous oxidative stress that could readily damage macromolecules and disrupt ATP production at membrane lipid domains [421,426,427]. This unique feature implies that melatonin may have an intrinsic modulatory effect over phase separation, not only in early but present-day organisms (Figure 1).

## 6. Conclusions

The physiological and pathological functions of biomolecular condensates in neurodegenerative disorders are shaped by powerful, complex, interdependent relationships between membraneless organelles, membranes/lipid rafts, ATP, RNA, and most of all, stress and its timely resolution. Melatonin’s intimate association with each of these decisive influencers may position the potent, ancient antioxidant as an important mediator of the phase separation of condensates in health and disease via principal ATP-dependent mechanisms including post-translational modifications and RNA m6A modifications (Figure 1). This novel theoretical review is presented with the intention to spur further research interest and exploration in the full, multi-faceted potential of melatonin in the regulation of biomolecular condensates that could provide solutions and answers to existing and future challenges and questions in this exciting and promising field of study.

## Abbreviations

3-OHM	3-hydroxymelatonin
Aβ	β-amyloid peptide
AD	Alzheimer’s disease
ADP	adenosine diphosphate
AICD	amyloid precursor protein intracellular domain
ALS	amyotrophic lateral sclerosis
ANT	adenine nucleotide translocator
APP	amyloid precursor protein
ASC	apoptosis-associated speck-like protein containing a C-terminal caspase recruitment domain
ATP	adenosine triphosphate
CL	cardiolipin
CYB5R3	NADH-cytochrome b5 reductase 3
Cyt	c cytochrome c
DDX3(X)	DEAD-box RNA helicase
DNA	deoxyribonucleic acid
eIF2a	eukaryotic translation initiation factor 2 alpha
ER	endoplasmic reticulum
FTO	frontotemporal dementia
FUS	fused in sarcoma
H+	proton
H_2_O_2_	hydrogen peroxide
IDP	intrinsically disordered protein
IDR	intrinsically disordered region
IMM	inner mitochondrial membrane
L_o_	liquid-ordered
L_c_	circular liquid-condensed
L_d_	liquid-disordered
LLPS	liquid–liquid phase separation
m^6^A	N^6^-methyladenosine
mM	millimolar
μM	micromolar
MDM2	mouse double minute 2 homolog
MLO	membraneless organelle
MAM	mitochondria-associated membrane
MOM	mitochondrial outer membrane
mPTP	mitochondrial permeability transition pore
mRNA	messenger RNA
MT	microtubule
NE	nuclear envelope
NFT	neurofibrillary tangles
NLRP3	NLR pyrin domain containing 3 (inflammasome)
nM	nanomolar
NPC	nuclear pore complex
O_2_^•−^	superoxide radical
^•^OH	hydroxyl radical
^•^OOH	hydroperoxyl radical
OXPHOS	oxidative phosphorylation
PD	Parkinson’s disorder
PDC	pyruvate dehydrogenase complex
PDK	pyruvate dehydrogenase kinase
Pi	inorganic phosphate
PLD	prion-like domain
PML	promyelocytic leukemia proteins
PTM	post-translational modification
RNA	ribonucleic acid
RBP	RNA-binding protein
RNP	ribonucleoprotein
ROS	reactive oxygen species
RP	ribosomal protein
SG	stress granule
SUMO	small ubiquitin-like modifier
TCA	tricarboxylic acid (cycle)
TDP-43	TAR DNA-binding protein 43
Ub	ubiquitin
UCP1	uncoupling protein 1
UPS	ubiquitin-protease system
VDAC	voltage-dependent anion channel
ZFP217	zinc finger protein 217
